# Interaction of the human erythrocyte Band 3 anion exchanger 1 (AE1, SLC4A1) with lipids and glycophorin A: Molecular organization of the Wright (Wr) blood group antigen

**DOI:** 10.1371/journal.pcbi.1006284

**Published:** 2018-07-16

**Authors:** Antreas C. Kalli, Reinhart A. F. Reithmeier

**Affiliations:** 1 Leeds Institute of Cancer and Pathology, University of Leeds, Leeds, United Kingdom; 2 Astbury Centre for Structural Molecular Biology, University of Leeds, Leeds, United Kingdom; 3 Department of Biochemistry, University of Toronto, Toronto, Canada; Fox Chase Cancer Center, UNITED STATES

## Abstract

The Band 3 (AE1, SLC4A1) membrane protein is found in red blood cells and in kidney where it functions as an electro-neutral chloride/bicarbonate exchanger. In this study, we have used molecular dynamics simulations to provide the first realistic model of the dimeric membrane domain of human Band 3 in an asymmetric lipid bilayer containing a full complement of phospholipids, including phosphatidylinositol 4,5–bisphosphate (PIP_2_) and cholesterol, and its partner membrane protein Glycophorin A (GPA). The simulations show that the annular layer in the inner leaflet surrounding Band 3 was enriched in phosphatidylserine and PIP_2_ molecules. Cholesterol was also enriched around Band 3 but also at the dimer interface. The interaction of these lipids with specific sites on Band 3 may play a role in the folding and function of this anion transport membrane protein. GPA associates with Band 3 to form the Wright (Wr) blood group antigen, an interaction that involves an ionic bond between Glu658 in Band 3 and Arg61 in GPA. We were able to recreate this complex by performing simulations to allow the dimeric transmembrane portion of GPA to interact with Band 3 in a model membrane. Large-scale simulations showed that the GPA dimer can bridge Band 3 dimers resulting in the dynamic formation of long strands of alternating Band 3 and GPA dimers.

## Author summary

Human Band 3 (AE1, SLC4A1), an abundant 911 amino acid glycoprotein, catalyzes the exchange of bicarbonate and chloride across the red blood cell membrane, a process necessary for efficient respiration. Malfunction of Band 3 leads to inherited diseases such as Southeast Asian Ovalocytosis, hereditary spherocytosis and distal renal tubular acidosis. Despite much available structural and functional data about Band 3, key questions about the conformational changes associated with transport and the molecular details of its interaction with lipids and other proteins remain unanswered. In this study, we have used computer simulations to investigate the dynamics of Band 3 in lipid bilayers that resemble the red blood cell plasma membrane. Our results suggest that negatively charged phospholipids and cholesterol interact strongly with Band 3 forming an annulus around the protein. Glycophorin A (GPA) interacts with Band 3 to form the Wright (Wr) blood group antigen. We were able to recreate this complex and show that GPA promotes the clustering of Band 3 in red blood cell membranes. Understanding the molecular details of the interaction of Band 3 with GPA has provided new insights into the nature of the Wright blood group antigen.

## Introduction

**Fig 1:**
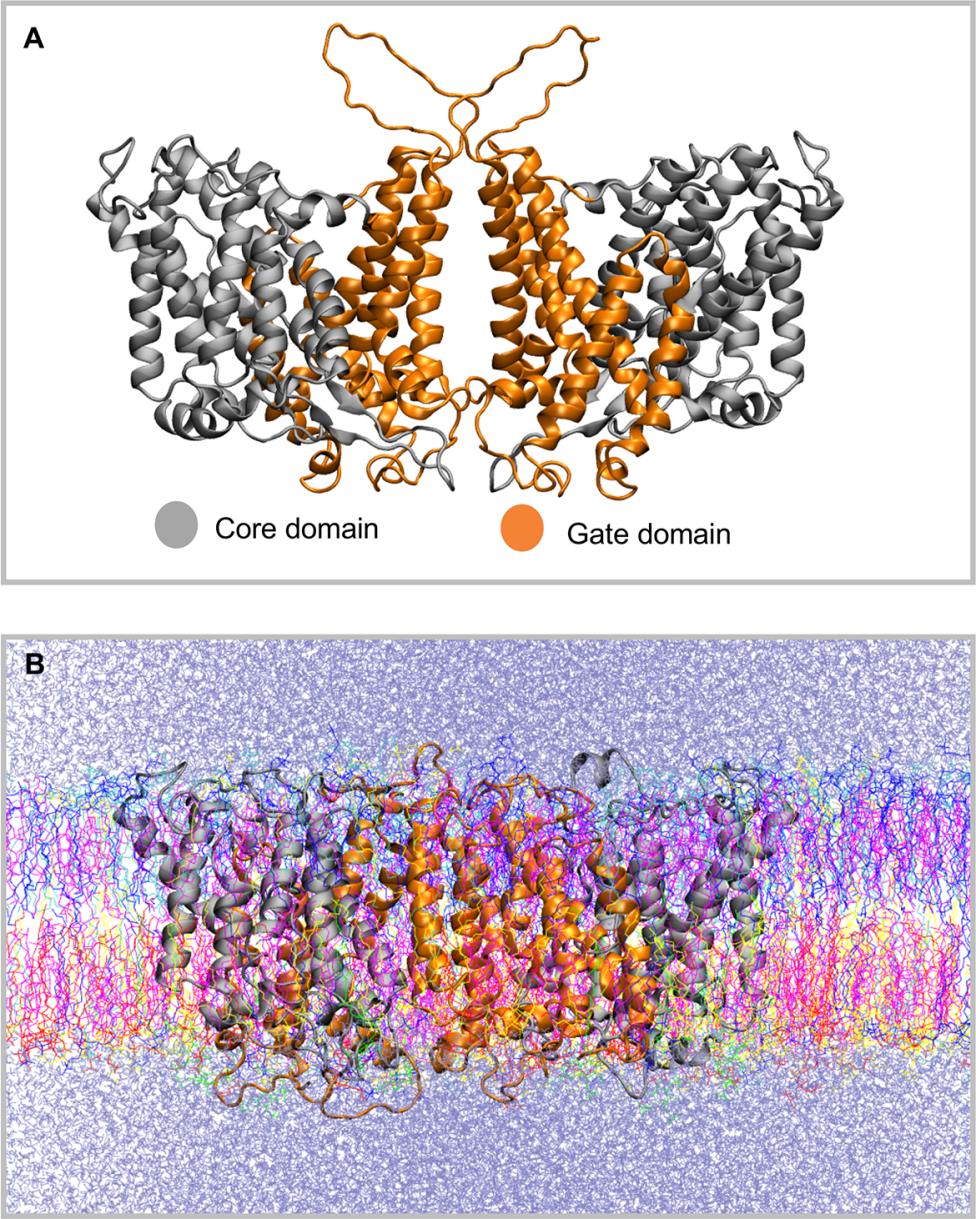
Band 3 structure. A. Structure of the dimeric Band 3 membrane domain (mdAE1) that was used in our simulations. The mdAE1 core domain is shown in grey and the gate domain in orange. Note that some unstructured regions that are missing from the crystal structure (PDB: 4YZF) have been modelled. B. Snapshot from the end of one of the atomistic simulations in which mdAE1 is embedded in a complex asymmetric bilayer (Band3_AT-1). The different lipid types are shown in different colors and the water is shown in ice-blue.

Band 3, the human erythrocyte anion exchanger 1 (AE1, SLC4A1) is responsible for the rapid electro-neutral exchange of chloride and bicarbonate across the plasma membrane; a process that increases the blood’s capacity to carry carbon dioxide as plasma bicarbonate [1]. Human Band 3 is a 911 amino acid glycoprotein consisting of a N-terminal cytosolic domain (cdAE1, residues 1–360) responsible for the interaction with the cytoskeleton [2] and a C-terminal membrane domain (mdAE1, residue 361–911) responsible for its transport function [3,4]. Band 3 is predominantly a dimer in the membrane and when isolated in detergent solutions [5,6]. The isolated cdAE1 is also a dimer [7] as is the mdAE1 [8–10]. The crystal structure of the human mdAE1 was reported in 2015 [11]. Each subunit consists of 14 transmembrane (TM) segments arranged in an inverted 7 + 7 topology (Fig 1) in agreement with most topology studies [12–20]. The bacterial UraA proton-coupled uracil transporter [21], a bacterial SLC26 fumarate transporter [22], and a fungal proton-purine symporter, UapA [23] have a similar 7 + 7 inverted repeat topology creating a novel class of seven transmembrane segment inverted repeat carriers [24]. Each monomer consists of two sub-domains with TM1, 2, 3, 4 and TM8, 9, 10, 11 coming together to form a core domain and TM5, 6, 7 and TM12, 13 and 14 to form a gate domain (Fig 1). It is the relative movement of these two sub-domains that provides the alternating access to the central anion binding site [25]. The molecular details of this movement have yet to be established, likely operating in rocker-switch or elevator mode [26–28]. The dimer is held together by a central 4-helix bundle with predominant interactions at the extra-cellular ends of TM5 and 6, however there is significant space in the dimer interface of the crystal structure. It is unclear why the 4-helix bundle is not more tightly packed or if the dimer interface is dynamic.

The native mdAE1 was produced by limited proteolysis of red cell ghosts prepared from intact erythrocytes pretreated with the anion transport inhibitor H_2_DIDS [29,30]. This inhibitor reacts covalently with Lys539 and Lys851 within the gate domain, crosslinking two parts of the protein together and locking it into an outward-facing conformation [31]. The presence of the inhibitor is known to stabilize the protein against thermal denaturation [32–34]. The crystals were formed using a Fab fragment [35] in the presence of dodecylmaltoside, a commonly-used detergent known to stabilize Band 3 [36,37]. While the recent crystal structure of mdAE1, albeit static, can provide significant new insights into the Band 3 function, it does not include its native lipid environment. One purpose of our simulations was to determine the structure of mdAE1 in the absence of antibody and the H_2_DIDS inhibitor in a lipid bilayer rather than in a detergent micelle. Similar MD simulations of the bacterial UraA uracil transporter revealed specific interactions with lipids and an intermediate closed state in the absence of substrate [38].

Additionally, it has been shown that Band 3 function may be regulated by lipids [39–46]. The second purpose of our simulations was to assemble the dimeric mdAE1 into an asymmetric lipid bilayer containing cholesterol and other phospholipids to mimic the native red cell membrane. Fluorescence digital imaging showed that Band 3 is localized to PC-rich domains of erythrocyte membranes and is excluded from PS-rich domains [47]. In the erythrocyte membrane, pyrene-labelled phosphoinositides preferentially associate with Band 3 compared to PC [48]. Reconstitution experiments showed that transport activity of purified AE1 is sustained in the presence of PC and PE, but is inhibited by enriched levels (beyond 30 mole %) of PS [39]. Head groups also affect Band 3 stability with PE and PC stabilizing the protein while acidic lipids PG and PS destabilizing the protein. EPR measurements on ghost membranes indicated a strong interaction of spin-labelled cholesterol with Band 3 [45]. Enriched levels of cholesterol in erythrocytes deceased anion transport activity perhaps by restricting the conformational change in Band 3 that occurs during transport [49]. Band 3 interacts strongly with cholesterol and is proposed to contain a high affinity inhibitory cholesterol-binding site [44,46]. No tightly-associated lipid or detergent molecules were however resolved in the 3.5Å crystal structure of mdAE1. While collectively all these data suggest that lipids play a major role in Band 3 function, the molecular details of the Band 3/lipid interactions remain largely unknown, a knowledge gap that can be filled by computational studies.

Glycophorin A (GPA) is known to interact with AE1 in the endoplasmic reticulum (ER) and to promote Band 3 trafficking to the cell surface [50–53]. Anion transport is impaired in red cells devoid of glycophorin A [54,55]. In the mature erythrocyte the GPA/Band 3 complex forms the Wright (Wr) blood group antigen [56]. This interaction involves the specific interaction of Glu658 in Band 3 with Arg61 in GPA [56]. A Glu658Lys mutation creates the Wrb antigen perhaps due to the dissociation of the complex, although this has not been established. In the mdAE1 structure Glu658 is located in the extracellular loop connecting TM7 and TM8 on the periphery of the structure far away from the dimer interface. Despite the fact that the structures of both the mdAE1 and TM portion of GPA are available, no structure of the mdAE1/GPA complex exists and also the molecular details of this complex or its dynamics are largely unknown.

Using MD simulations, we created a dynamic model of mdAE1 in a native membrane and studied its interaction with lipids, and with GPA. We discovered that certain basic residues had specific interactions with inner leaflet acidic phospholipids phosphatidylserine and PIP_2_. Cholesterol was also enriched in the annular lipids surrounding the protein, but also at the dimer interface. We demonstrate that GPA association with mdAE1 occurs not only via an interaction of Arg61 with Glu658 but also via additional interactions of the TM and extracellular part of GPA with Band 3. Finally, large-scale simulations showed that the GPA dimer could sequentially bridge mdAE1 dimers creating alternating strands of these two proteins in a lipid bilayer. The MD simulations provide the first dynamic model of Band 3 in a complex lipid bilayer and molecular details about the interaction of Band 3 with lipids and with GPA.

## Results

### Interaction of Band 3 with anionic lipids

**Fig 2:**
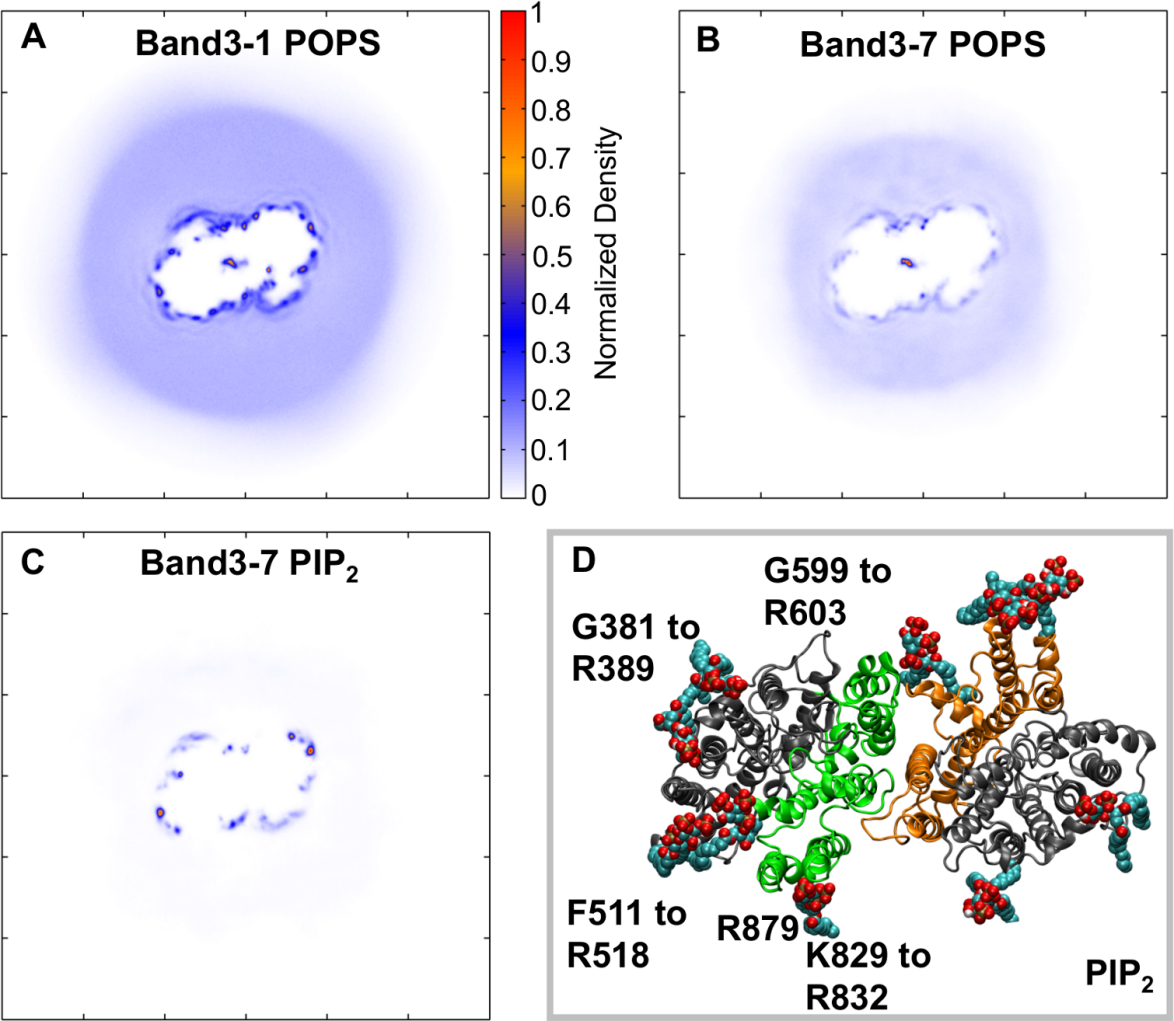
Interaction of Band 3 with anionic lipids. A,B,C. Two-dimensional density of POPS (A, B) and PIP_2_ (C) molecules around Band 3 in the bilayer inner leaflet. The density in A was calculated using all repeat simulations of the Band3-1 (i.e. without PIP_2_) system and the densities in B and C were calculated using all repeat simulations of the Band3-7 system (see Table 1). D. Snapshot from the end of one of the atomistic simulations showing the interactions of mdAE1 dimer with PIP_2_ lipids. See also S1 and S2 Figs.

To study the interaction of the mdAE1 dimer with the different lipids that are present in the erythrocyte plasma membrane, the 1-palmitoyl-2-oleoyl-phosphatidylcholine (POPC) lipids that were self-assembled around mdAE1 (see [1]) were exchanged with other lipids using a computer script [57] to create an asymmetric bilayer containing 45% POPC and 5% 1-palmitoyl-2-oleoyl-phosphatidylethanolamine (POPE) in the outer leaflet and 12% POPC, 23% POPE, 15% 1-palmitoyl-2-oleoyl-phosphatidylserine (POPS) in the inner leaflet. Note that in these simulations the H_2_DIDS molecule present in the mdAE1 crystal structure was removed, as was the antibody used for crystallization. Five independent simulations of 10 μs each were performed. Analysis of the lipid density around the protein revealed a high density of POPS lipids around mdAE1 (Fig 2A). Indeed, during the simulations a discontinuous anionic annulus is formed around mdAE1 in the inner bilayer leaflet. Analysis of the interactions between mdAE1 and POPS lipids showed that the POPS head groups interact predominantly with positively charged Lys and Arg residues on the mdAE1 surface. The residues that form the highest number of contacts are at positions R387, R514, K600, K826, T830, W831, R832, H834 and R879.

To study further the interactions between lipids and mdAE1 and how this may be affected by other lipids, including PIP_2_ and cholesterol, we have also run simulations of mdAE1 in increasing complex bilayers. In particular, we have constructed seven different bilayers of increasing complexity with lipids that are found in the red blood cell membrane (see Table 1). In all cases the anionic annulus around mdAE1 was retained. The contact positions of PIP_2_, POPS and POPE around Band 3 are shown in S1 Fig. The preferential interactions of POPS lipids were reduced in certain areas around mdAE1 when PIP_2_ lipids were present in our simulations (Fig 2B and 2C). The red blood cell plasma membrane contains a small percentage (~2%) of PIP lipids in the inner leaflet. Despite the small number of PIP_2_ molecules in our simulation, the discontinuous anionic annulus around mdAE1 was formed mainly by PIP_2_ lipids and not POPS (Fig 2). Additionally, the PIP_2_ lipids were more specifically-localized in their interactions compared to POPS lipids. PIP_2_ molecules mainly interacted with the regions comprised of residues G381 to R389, R514, G599 to R603, H703, K826, K829 to R832, and R879. The dimeric nature of mdAE1 allowed the comparison of the lipid interactions in the two subunits, providing a built-in replicate. Indeed, both subunits exhibited similar pattern of lipid interactions (Fig 2).

**Table 1:** Summary of simulations with Band 3.

Simulation	Composition Outer leaflet	Composition Inner leaflet	Duration
*Coarse-grained*:			
*Band3-1*	POPC:POPE (~45:5)	POPC:POPE:POPS (~12:23:15)	5 x 10 μs
*Band3-2*	POPC:POPE:CHOL (~42.5:5:2.5)	POPC:POPE:POPS:CHOL (~11.5:21.5:14.5:2.5)	5 x 10 μs
*Band3-3*	POPC:POPE:CHOL (~41:4:5)	POPC:POPE:POPS:CHOL (~11:20:14:5)	5 x 10 μs
*Band3-4*	POPC:POPE:CHOL (~30:7.5:12.5)	POPC:POPE:POPS:CHOL (~9.5:16:12:12.5)	5 x 10 μs
*Band3-5*	POPC:POPE:CHOL (~22.5:2.5:25)	POPC:POPE:POPS:CHOL (~6:11.5:7.5:25)	5 x 10 μs
*Band3-6*	POPC:POPE:CHOL (~22.5:2.5:25)	POPC:POPE:POPS:PIP_2_:CHOL (~6:11.5:6.5:1:25)	5 x 10 μs
*Band3-7*	POPC:SM:POPE:CHOL (~11.5:11:2.5:25)	POPC:SM:POPE:POPS:PIP_2_:CHOL (~4:2:11.5:6.5:1:25)	5 x 10 μs
*Band3-8*	POPC:SM:POPE:CHOL (~21.5:21:5:2.5)	POPC:SM:POPE:POPS:CHOL (~7.5:4:21.5:14.5:2.5)	5 x 10 μs
*Atomistic*:			
*Band3_AT-1*	POPC:SM:POPE:CHOL (~11.5:11:2.5:25)	POPC:SM:POPE:POPS:PIP_2_:CHOL (~4:2:11.5:6.5:1:25)	3 x 250 ns
*Band3_AT-2*	POPC:POPE (~45:5)	POPC:POPE:POPS (~12:23:15)	3 x 250 ns

The strong interactions of PIP_2_ molecules with mdAE1 also reduced the diffusion of the PIP_2_ molecules compared to the other lipids in our systems. In the Band3-6 (Table 1) simulations in which PIP_2_ molecules were part of the bilayer, the diffusion coefficient of all PIP_2_ lipids is 0.51 ± 0.07 x10^-7^ cm^2^/s (linear diffusion). The diffusion coefficients for POPC, POPE and POPS lipids is 1.28 ± 0.06 x10^-7^ cm^2^/s, 1.04 ± 0.06 x10^-7^ cm^2^/s, 0.92 ± 0.09 x10^-7^ cm^2^/s, respectively. The reduced diffusion of PIP_2_ lipids possibly is due to their strong interactions with the protein. A similar trend occurs in the Band3-7 simulation in which we have PIP_2_ lipids and sphingomyelin. We note that the diffusion of all lipids reduces as we increase the concentration of cholesterol. For example, the POPC diffusion in the system with no cholesterol is 5.89 ± 0.06 x10^-7^ cm^2^/s, compared to 1.28 ± 0.06 x10^-7^ cm^2^/s in the systems we have the native 50% level of cholesterol.

### Cholesterol interactions with AE1

**Fig 3:**
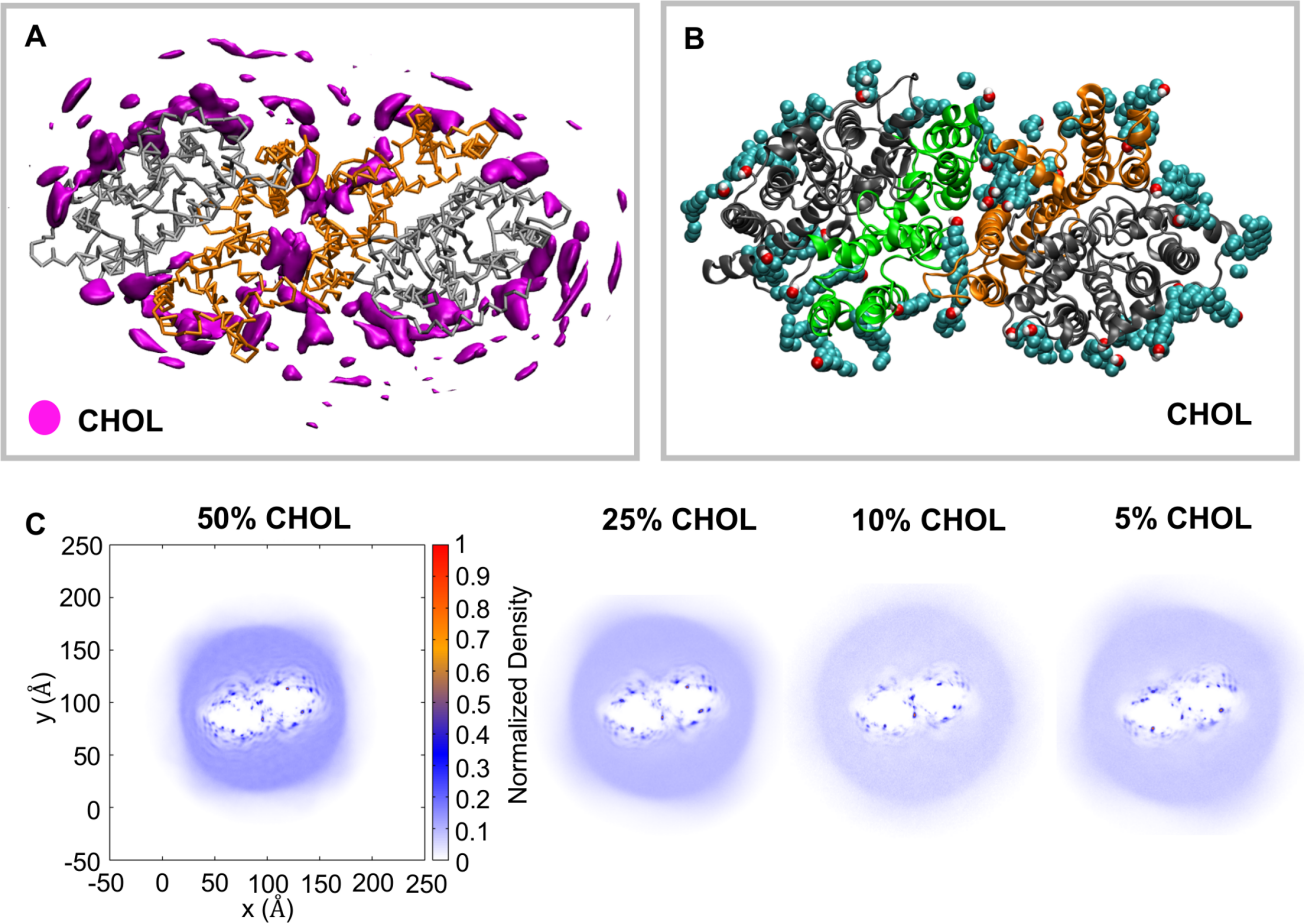
Interactions of Band 3 with cholesterol. A. Average density of cholesterol around mdAE1. The density was calculated using all repeat simulations of the Band3-7 system (see Table 1). B. Snapshot from the end of one of the simulations showing the interactions of mdAE1 dimer with cholesterol. C. Two-dimensional density of the cholesterol around mdAE1. Different systems are shown that contain different concentration of cholesterol. The normalization was done by dividing the number of lipids in each bin with the number of frames and the bin area. After the density calculation, the density was divided by the largest density and thus the highest density region has a value of 1. See also S1 Fig.

The red blood cell plasma membrane consists of a high concentration of cholesterol (~50% of lipids), which may regulate the function of AE1. To study the interactions of Band 3 with cholesterol we have run simulations of the mdAE1 dimer in bilayers with increasing concentration of cholesterol (5%, 10%, 25% and 50%). Cholesterol showed a preferential interaction with mdAE1 regardless of its concentration in the membrane (Fig 3). Increasing the cholesterol concentration increased the number of cholesterol molecules surrounding mdAE1. Analysis of the radial density of cholesterol showed an enrichment of cholesterol in most parts of the annular layer surrounding mdAE1 at 5% cholesterol and this enrichment did not change upon increasing the cholesterol concentration to 10%, 25% and 50%. Interactions of cholesterol with mdAE1 were rather dynamic and cholesterol could be seen to “flip-flop” across the membrane during the simulation, a process observed with native erythrocytes [58]. Analysis of the interactions between the cholesterol hydroxyl group and mdAE1 suggested residues V383, F401, S438, I442, F515, F544, K600, I624, L775, H834 and R871 made the largest number of interactions with the cholesterol hydroxyl head group (Fig 3 and S1D Fig). Interestingly, cholesterol is also bound in the dimer interface (Fig 3A and 3B). In the mdAE1 crystal structure there is a cavity in the dimer interface that in our simulations is filled mainly with cholesterol.

### Atomistic simulations

In order to study the interactions between the lipids and mdAE1 in molecular detail, snapshots from 3 different coarse-grained simulations (from the Band3-7 system; Table 1) were converted to atomistic representations and further simulations of 250 ns were performed. The conversion was done as described in [59]. Calculation of the interactions between PIP_2_ and mdAE1 suggests that the interactions observed in our coarse-grained simulations above were retained (S3A Fig). In the atomistic simulations, residues L382 to R384, R387, K600 and K829 to R832 made the highest number of interactions with PIP_2_ lipids. Cholesterol was bound at sites that commonly contained an aromatic residue. In the atomistic simulations, the polar hydroxyl group of cholesterol made significant interactions with residues Y390, Y393, A400 to S402, F511, F515, Q545, K600, W648, W662 and H834 (S3B Fig).

**Fig 4:**
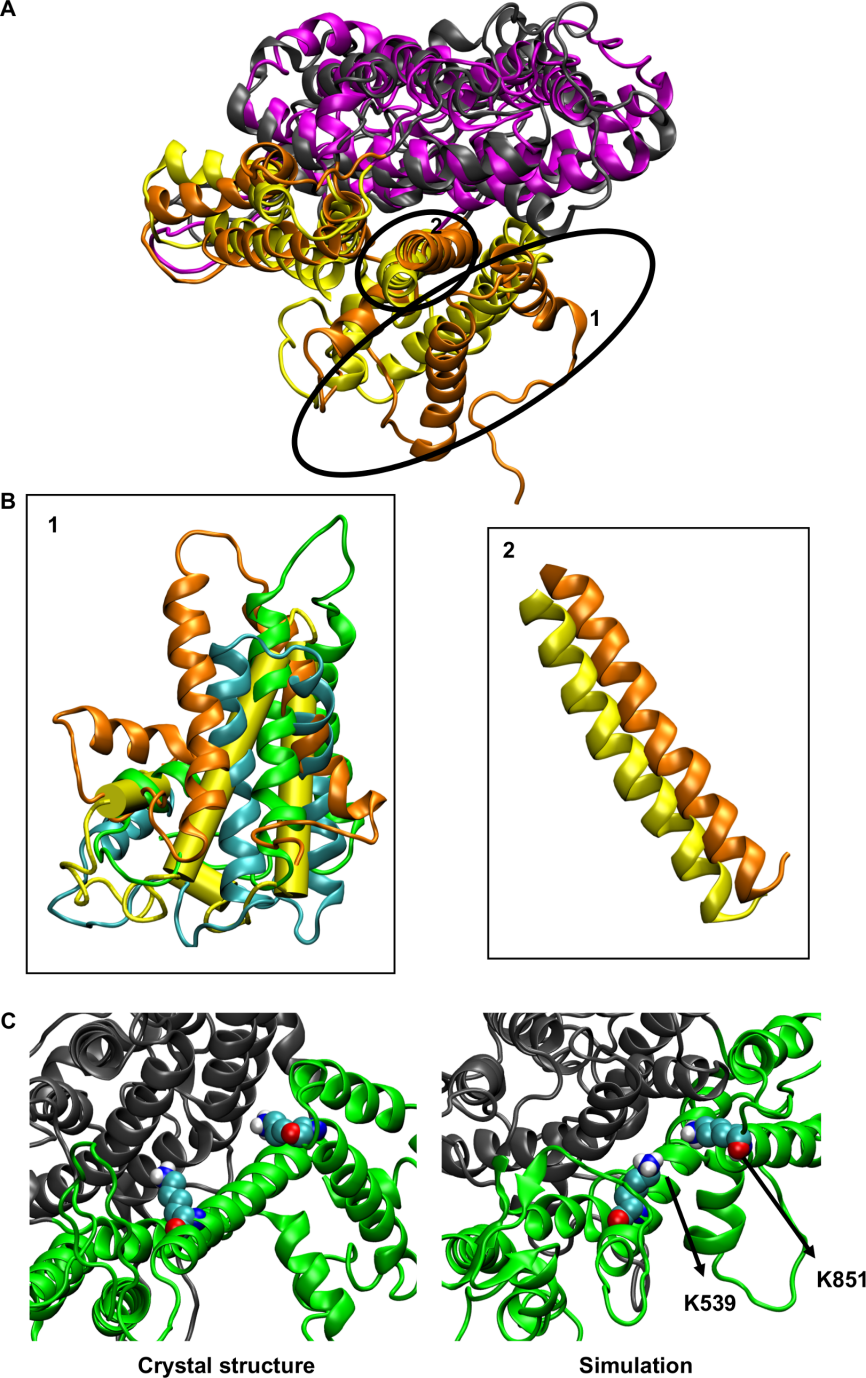
Atomistic simulations of Band 3. A. Alignment of the helical regions of the core domain, with the exception of helix 3, of a Band 3 monomer from Band3_AT-1 simulations. The variation in the position of helices 13, 14 and H6 and the change in the position of helix 5 are shown in B. The gate domain of the crystal structure is shown in yellow. C. Position of the K539 and K851 in the mdAE1 crystal structure and at the end of one of our simulations.

Comparison of the protein structures at the end of our atomistic simulations with the mAE1 crystal structure reveals a significant fluctuation in the position of TM helices 13, 14 and H6. The movement of those helices varied between the simulation repeats (Fig 4A and 4B). Additionally, a small shift of helix 5 of the gate domain towards the core domain is observed in most simulations (Fig 4B). Interestingly, no major changes within the core domain were observed with the exception of helix 3, which shifts towards the gate domain. These changes in the helices resulted in structures that in most simulations are still in an outward-open conformation but the opening in the extracellular region is narrower. We note that some of the changes in the position of helices 13, 14 and H6 were observed during the coarse-grained simulations. Additionally, in some of the simulations, some of the helicity of H1 is lost. Similar changes within the domains were observed in atomistic simulations in which snapshots of the Band3-1 systems (i.e. without any cholesterol) were performed. In this case however, a change in helix 3 position was observed only in one of the simulations.

In the crystal structure H_2_DIDS crosslinked K539 in TM5 to K851 that is located at the end of helix 13. The side chain of K851 still points towards the core domain in our simulations, however in the majority of the simulation it is closer to K539, the H_2_DIDS reactive lysine. In particular, the minimum distance between K539 and K851 in the crystal structure is ~1.5 nm while at the end of the atomistic simulation (Band3_AT-1 system) the same distance is reduced to 0.77 ± 0.23 nm (Fig 4C). The Cα distance between the same residues in the crystal structure is ~1.9 nm. In our simulations, the K539 Cα/K851 Cα distance varies between ~1 nm to ~1.5 nm because of the variation in the position of helices 13 and 14 discussed above.

**Fig 5:**
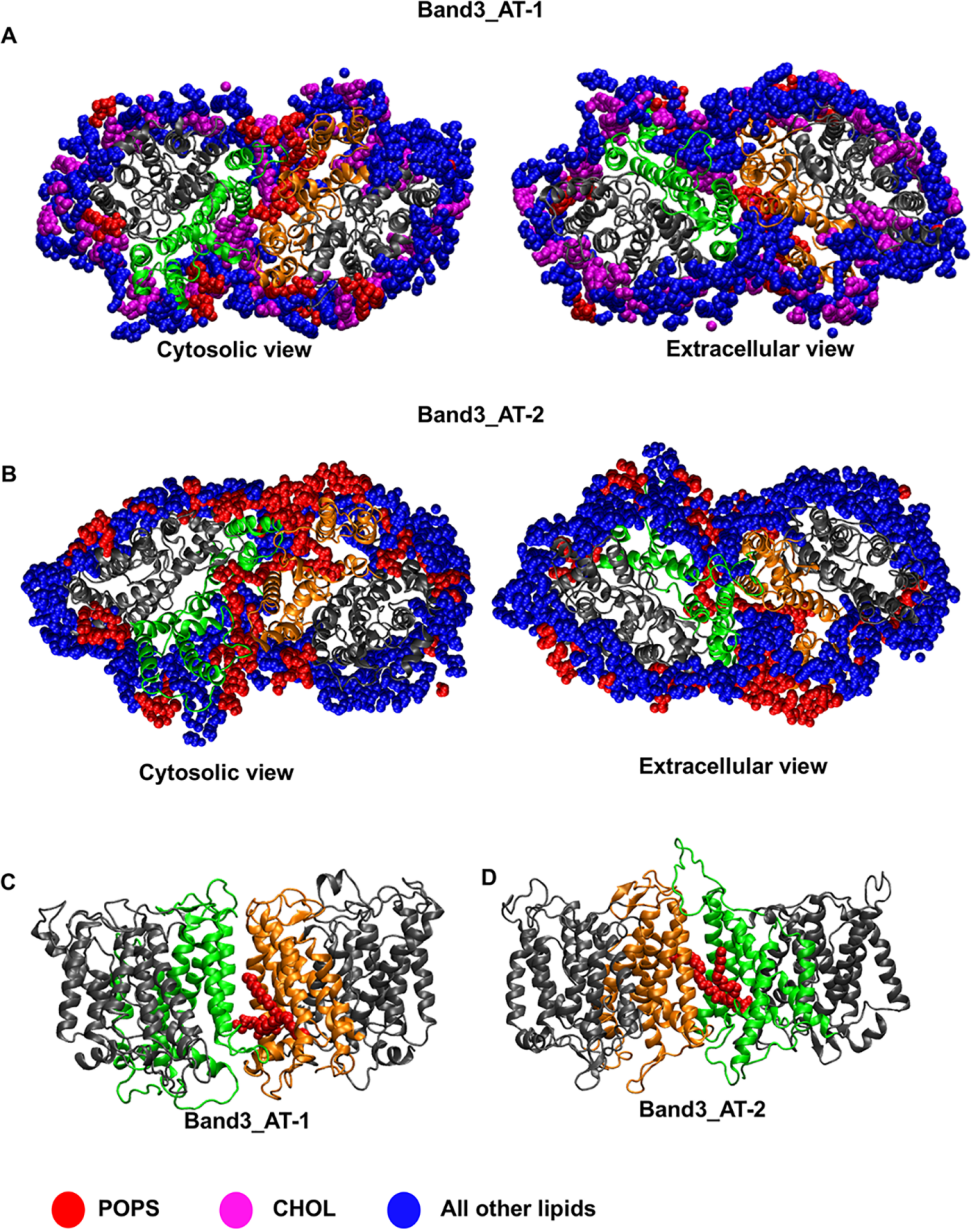
Lipids in the Band 3 dimer interface. A, B. Lipids around the mdAE1 dimer at the end of one of the Band3_AT-1 (A) and Band3_AT-2 (B) simulations. Cholesterol is shown in magenta, POPS in red and the rest of the lipids are shown in blue. C, D. The POPS lipid found between the two monomers of mdAE1 is our simulation is shown for the Band3_AT-1 (C) and Band3_AT-2 (D) simulations. Note that in this Figure we show only the lipids that are in immediate vicinity of the protein. See also S3 Fig.

The crystal structure of the mdAE1 dimer revealed a rather loose packing of the two monomers. Using the trj_cavity program [60] we have estimated that the cavity between the two mdAE1 monomers is 7228 Å^3^. Analysis of the lipid distribution in all our AT simulation suggests that the space between the two dimers in the crystal structure was filled with cholesterol and with the lipid tails of the other phospholipids, mainly the POPS lipids in the inner leaflet (Fig 5). This suggests that cholesterol and other lipids can regulate the interaction between the Band 3 monomers. A POPS lipid can be seen in the simulation without any cholesterol in the bilayer; this lipid was also in the interface in the coarse-grained simulations (Fig 5C). The rest of the space in this case is filled with other lipid tails. The head group of the POPS lipid interacted mainly with lysines and arginines at the cytoplasmic end of helix 5. In the simulations with 50% of cholesterol the space between the two monomers is filled mainly with cholesterol molecules and a single POPS lipid (Fig 5). Interestingly, calculation of the interactions between the two monomers during the atomistic simulations showed that the interactions that were observed in the crystal structure were mostly retained (S4 Fig). In the simulations, additional interactions between residues in region L573 to S595 in the two monomers that are not observed in the crystal structure occurred, allowing the dimer to somewhat optimize its packing. These interactions were higher in the simulations without any cholesterol in the bilayer (S4 Fig). At the end of the simulations with 50% cholesterol the volume of the cavity between the mdAE1 monomers was estimated to 7874 ± 815 Å^3^. This is close to the volume of the crystal structure (7228 Å^3^) suggesting that the presence of cholesterol retained the cavity between the two monomers. In contrast, the volume of the cavity in the simulations without any cholesterol in the bilayer was 5595 ± 675 Å^3^ suggesting that whilst there is still a cavity between the monomers (because of the presence of the lipid tails) this is smaller compared to the crystal structure.

### GpA/Band 3 interactions

**Fig 6:**
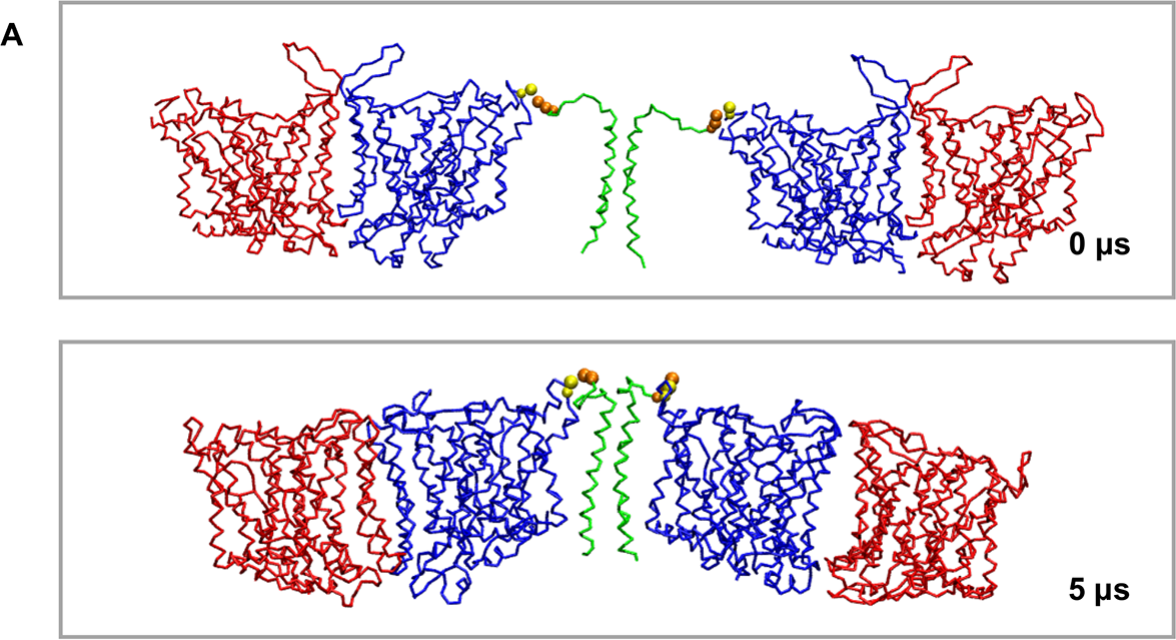
Interactions of Band 3 with GPA. A. Position of the Band 3 dimers and of the GPA dimer at the beginning and at the end of one of the simulations with the Band 3 and GpA dimers. The Band 3 monomers are shown in red and blue and the GPA in green. The GPA Arg61 and Band 3 Glu658 residues that were restrained to interact in one of our simulation systems are shown in yellow and orange VDW, respectively. See also S5, S6, S7, S8 and S9 Figs.

As mentioned above, Band 3 interacts directly with GPA and this interaction is very important for the trafficking of Band 3 to the cell surface. Despite many functional studies, there is limited structural data for the Band 3/GPA complex. This is partly due to the weak nature of this interaction that makes it challenging to obtain structural information for the nature and dynamics of this complex. To study this complex, we have added the dimeric (residues Arg61-Lys101) transmembrane helical region of the GPA [61,62], and two mdAE1 dimers in a complex bilayer that resembles the native red blood cell plasma membrane (Fig 6A). The structure of GPA [61] revealed a dimeric structure held together by TM helix-helix interactions. Furthermore, it has been shown that Arg61 located in the extracellular unstructured region of GPA interacts directly with Glu658 at the loop connecting TM7 and TM8 of Band 3 to form the Wright blood group antigen [56]. The TM helical part of GPA in our simulations extends from Ile73 to Arg96. The mdAE1/GPA/mdAE1 complex was modelled so that GPA is at the greatest distance from mdAE1 possible with this ionic interaction restrained during the simulation (see Fig 6A). We have performed 5 simulations of 5 μs each with one GPA dimer and two mdAE1 dimers (Table 2). Interestingly, in our simulations the transmembrane segments of the GPA dimer interact with the two mdAE1 dimers, forming a bridge between the two Band 3 dimers (Fig 6A). Analysis of the interactions between GPA and mdAE1 in all simulations revealed that GPA interacts with some residues in the following regions of mdAE1: i) Thr481 to Leu484 (top of TM helix 3 and loop connecting TM helices 3 and 4), ii) Val488 to Phe511 (TM helix 4 and the beginning of parallel helix H2), and iii) Trp648 to Phe665 (region connecting TM helices 7 and 8 and top of helix 8). In some of the simulations the GPA also interacts with mdAE1 residues Gly381 to Arg384 at the beginning of the parallel helix H1 or with Ala666 to Leu672 residues (TM helix 8; Fig 6 and S5 and S8 Figs). As shown in S8A Fig GPA interacts with Band 3 with residues that face away from the GPA interface. Alignment of the mdAE1/GPA complexes (10 complexes) at the end of the Band3/GPA-1 simulation system suggests that while the GPA TM region interacts with the regions described above it can adopt somewhat modified positions/orientations in some of the simulations.

**Table 2:** Summary of simulations with Band 3 and GPA.

Simulation	Proteins	Duration
*Band3/GpA-1*	mdAE1/GPA with Glu658-Arg61 restrained	5 x 5 μs
*Band3/GpA-2*	mdAE1/GPA with no Glu658-Arg61 restrains	5 x 5 μs
*Band3/GpA-3*	mdAE1 Glu658Lys/GPA	5 x 5 μs
*Band3-large*	mdAE1	1 x 10 μs
*Band3/GpA-large*	mdAE1/GPA complex	1 x 10 μs

When the same simulations were performed but without the restraints on the mdAE1 Glu658/ GPA Arg61 interaction, in all simulations GPA interacted in a similar fashion as above but with only one of the mdAE1 dimers (S5C, S5D and S8 Figs). The second mdAE1 dimer diffused away in the bilayer (S6 Fig). This is perhaps expected at this timescale because even in the simulation in which the mdAE1 Glu658-GpA Arg61 interaction was restrained, GPA always interacted first with one of the mdAE1 dimers and after some time it interacted with the other one (S6A Fig). In the simulations where the mdAE1 Glu658-GpA Arg61 interaction is not restrained, GPA Arg61 interactions with Band 3 are rather dynamic. For most of the time, GPA Arg61 interacts with mdAE1 Glu658 but occasionally it interacts with other residues close to 658 or it does not interact with Band 3 at all (S6C and S6D Fig).

To examine how breaking the mdAE1 Glu658/GpA Arg61 interaction would affect the mdAE1/GPA complex formation we have mutated the Glu658 to Lys. This mutation will reverse the charge of residue 658 and will also mimic the Glu658Lys Wright blood group antigen variant. In these simulations, the interaction between Band 3 Lys658/ GpA Arg61 is no longer retained (S7 Fig). However, the helical TM portion and some of the extracellular part of GPA still interacts with the Band 3 TM region in a similar fashion to the simulations above with the wild type Band 3 (S7 Fig). The Glu658Lys mutation increases the dynamics of the GPA extracellular unstructured region comprised of residues 61 to 65 but the Band3/GPA TM complex can be formed due to the interactions of the GPA helical portion and of residues 66 to 73 that are adjacent to the GPA helical portion with Band 3. In the presence of the Glu658Lys mutation, GPA residues H66, H67, and F68 form the main interactions with Band 3 in the extracellular unstructured part of GPA. Thus, the Wright blood group antigen is probably due to a local conformational change in the epitope rather than complete dissociation of the GPA/Band 3 complex.

Calculation of the interactions of lipids with the GPA-mdAE1 complex reveals the same pattern of interactions as seen above for mdAE1 alone (S9A and S9B Fig). Additional interactions between the cluster of basic residues (Arg96, Arg97, Lys100 and Lys101) on the cytosolic site of GpA and PIP_2_ lipids were observed (S9C Fig). Cholesterol is still found in the interface of the two mdAE1 monomers in both dimers of the mdAE1-GPA-mdAE1 complex. Interactions between the cholesterol head group and GpA residues Ser92, Tyr93 and Arg96 to Ile99 in the cytosolic region are also observed (S9 Fig). Preferential interactions of GPA with cholesterol were also shown in other simulation studies [63]. Interestingly, in all systems the cavity in the dimer interface of both mdAE1 was empty at the beginning of our simulations (not occupied by lipids before the equilibration step). In the two systems that contained WT Band 3 (i.e. Band3/GpA-1 and Band3/GpA-2 systems), at the end of the simulations (after equilibration and 5 μs of simulation) the region in the dimer interface of one of the mdAE1 is occupied by a POPC lipid and cholesterol. The same region of the second mdAE1 is occupied only by cholesterol. In the systems with the mutated mdAE1, in addition to cholesterol one POPC was found in the interface of one of the mdAE1 dimers and three POPC lipids in the interface of the second dimer. This augments our previous observation that the region between the dimer is filled with lipids and cholesterol.

### Large-scale simulations of Band 3 and of Band3/GPA complex

**Fig 7:**
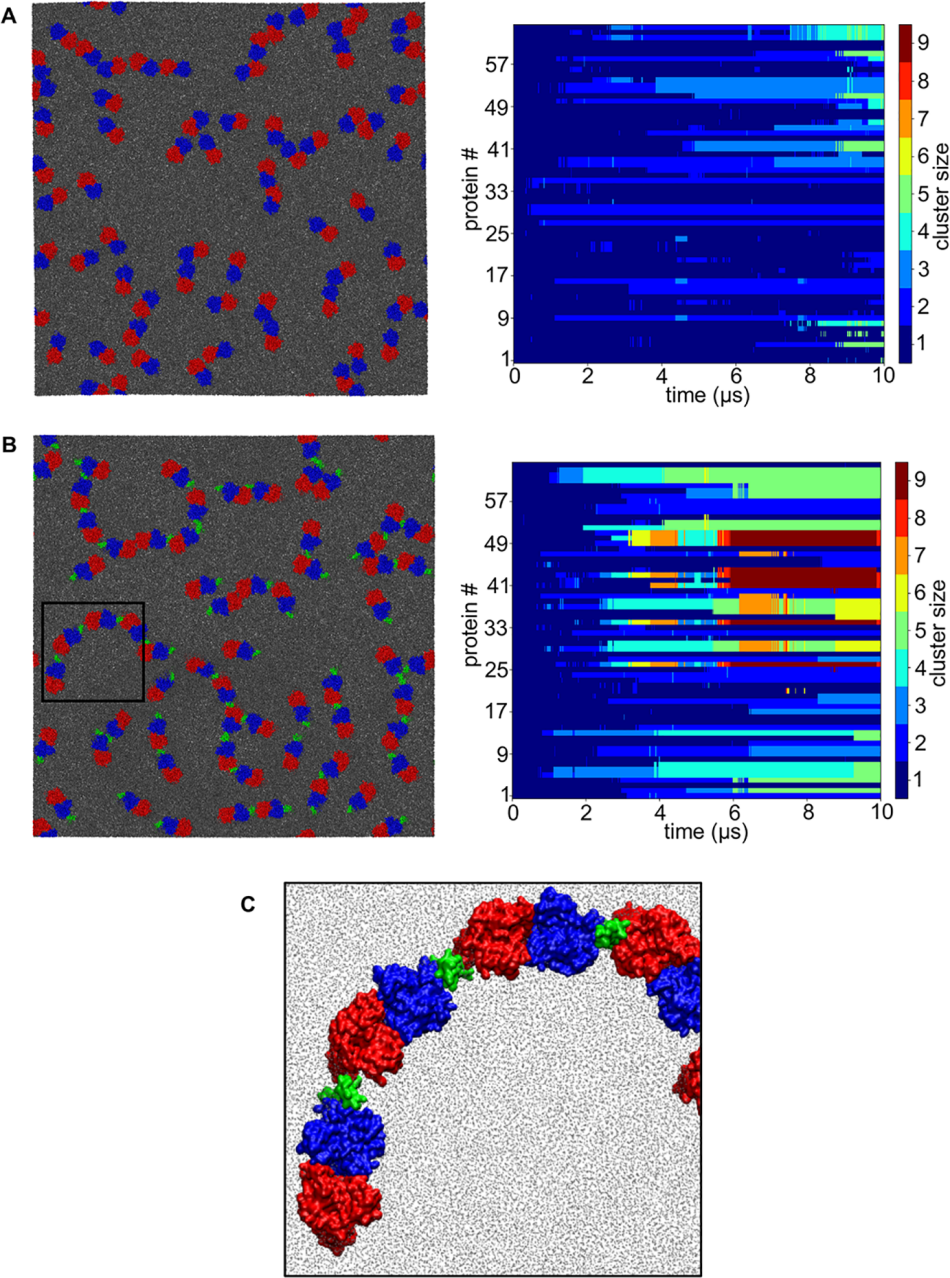
GPA promotes Band 3 clustering. A,B. Final snapshot of the simulations with the mdAE1 dimer and mdAE1/GPA complexes. The mdAE1 monomers are shown in red and blue and the GPA in green. Next to each simulation snapshot we show the clustering of proteins during the simulation as a function of time. Each line represents one protein in our simulation system and each color shows the association of each protein with a specific cluster. C. Zoom-in a region of the simulation in B showing the alternating arrangement of the mdAE1/GpA complex. See also S10 Fig.

Collectively our results above suggest that GPA may promote the clustering of Band 3 dimers. Given that in the Band 3 dimer the two gate domains interact with each other to form the dimer it is possible that a homodimer like GPA may mediate the interactions between the core domains of Band 3 dimers. To test further our observations above we have constructed two large-scale coarse-grained systems with the 64 Band 3 dimers alone or 64 Band 3/GPA complexes inserted in a POPC bilayer (S10A and S10B Fig). We note that in the Band 3/GPA simulation the complex was restrained with an elastic network and therefore no changes in the relative orientation/interactions between Band 3 and GpA were possible. For these simulations we have used the most frequent observed Band 3/GPA complex. In these simulations, the proteins cover ~20% of the surface area to mimic the physiological concentration of Band 3 in red blood cell membranes. We have simulated these two systems for 10 μs. At the end of the simulation with Band 3 alone, the largest cluster contained 5 dimers but approximately ~45% of the Band 3 dimers in this simulation were not part of a cluster (monomeric) and further ~25% of the Band 3 dimers were part of a cluster with two mdAE1 dimers (dimeric; Fig 7 and S10 Fig). In these simulations, Band 3 dimers interact mainly via residues in regions 478 to 484 in the extracellular loop connecting TM3 and 4, 492 to 507 in TM4, 658 to 670 in TM8 and 857 to 877 in TM14. In contrast, at the end of the simulation with the Band 3/GPA complexes the largest cluster consisted of 8 proteins (during the simulation the largest cluster consisted of 11 proteins) but in this case only ~20% of the Band 3/GPA complexes are not part of a cluster (compared to ~45% in the simulations with Band 3 alone; S10 Fig). Additionally, in the simulation with the Band 3/GPA complexes ~45% of the proteins were part of a cluster that had 5 or more proteins. In this simulation, the interaction between Band 3 dimers is mediated in most cases via the GPA as predicted in our simulations above and most of the time Band 3 and GPA are in a linear arrangement in good agreement with our simulations above (Fig 7). We also note that in the simulations with the Band 3/GPA complexes, clusters with four proteins or more were formed after approximately 2 μs of simulation whereas in the simulation with the Band 3 alone similar clusters (with 4 proteins or more) were formed after approximately 7.5 μs of simulation. Calculation of the anomalous diffusion of the two simulation systems showed that in the Band 3 simulation the diffusion coefficient was 4.08 ± 0.32 x10^-7^ cm^2^/s (scaling exponent of the non-linear fit value was 0.85 ± 0.01) whereas the diffusion coefficient of the Band 3/GPA complex was 3.24 ± 0.25 x10^-7^ cm^2^/s (scaling exponent of the non-linear fit value was 0.84 ± 0.01). Collectively, our results suggest that the presence of GPA promoted the clustering of Band 3 in red blood cell membranes.

## Discussion

In this study, we have created the first realistic atomistic model of mdAE1 in a complex lipid bilayer that mimics the native red cell membrane. Our MD simulations have shown preferential interaction of acidic lipids (POPS and PIP_2_) on the inner leaflet of the bilayer with specific sites on mdAE1. POPS has been shown to destabilize AE1 [42] and reduce its transport activity [39]. Thus, this acidic lipid may play a role in regulating the transport activity of Band 3 by binding to particular sites in the protein.

Phosphatidylinositol-4-phosphate kinase is associated with the human red cell membrane [64] and its product, PIP_2_ is involved in modulating Protein 4.1 interactions with erythrocyte membrane proteins, inhibiting binding to Band 3 and enhancing binding to Glycophorin C [65,66]. We found that PIP_2_ binds preferentially to specific sites on mdAE1. The high content of Band 3 in the erythrocyte membrane would sequester the bulk of this lipid to an annulus around Band 3 providing a “hot spot” for protein interactions and signaling.

We found that cholesterol interacts directly with the surface of mdAE1, an interaction that involves specific tryptophan residues. The human β2-adrenergic receptor was co-crystallized with a tightly-bound cholesterol molecule involving a close interaction with a tryptophan residue that stabilizes the protein [67,68]. Cholesterol binding sites were also identified at the mdAE1 dimer interface, suggesting that cholesterol may play a role in stabilizing or modulating the interaction between mdAE1 subunits. A number of biophysical studies have supported the view that cholesterol interacts with Band 3 and affects its dynamics, self-association and transport activity. It has been shown that cholesterol affects the aggregation state of Band 3 [69]. Band 3 has a high-affinity inhibitory cholesterol binding site [44]. A strong interaction of spin-labelled cholesterol with Band 3 has also been demonstrated [45]. Enriching cholesterol in red blood cells inhibits transport, while depleting cholesterol enhances transport [49]. Thus, cholesterol interaction with Band 3 modulates its transport activity, likely by rigidifying the dimer interface.

It has been suggested that cholesterol interacts at distinct binding sites in membrane proteins that consist of CRAC, CARC or tilted domains containing tyrosine residues [70]. The CRAC domain has a signature sequence L/V-X1-5-Y-X1-5-K/R while the CARC has the opposite orientation. MdAE1 in our simulations contains 14 tyrosine residues, of which 12 have basic residues +/- 5 residues away. Note, however, that Tyr 390/392/393, and Tyr 553/555 are very close to each other. The basic residue interacts with the hydroxyl group, the tyrosine with the cholesterol rings, and the Leu/Ile/Val with the isooctyl chain. TM5 contains the “CRAC” sequence–ISL**I**FI**Y**^534^ETFS**K**- at its C-terminal end. The isoleucine and tyrosine are on the same side of TM helix 5 facing the bilayer, however Lys539 is the H_2_DIDS reactive lysine facing the inhibitor-binding site. Interestingly in our coarse-grained simulation we observe the highest number of interaction with cholesterol close to Tyr 390, Tyr 413 and Tyr 534.

Band 3 exists in three populations in human erythrocytes: 1) tetrameric Band 3 associates with GPA, Ankyrin, protein 4.2 and the Rh complex in the Ankyrin-associated complex, 2) the junctional complex of spectrin, actin and protein 4.1, and 3) a freely mobile dimer fraction [71]. There is also considerable evidence that Band 3 and GPA interact in the red cell membrane [72]. The Wright blood group antigen involves the direct interaction of Arg61 on the extracellular part of GPA with Glu658 in the extracellular loop connecting TM7 and TM8 in Band 3 [56] and anti Wra antibodies can co-immunoprecipitate GPA and Band 3 [73]. In Band 3 knockout mice, GPA is absent [74]. Biophysical evidence [75–77] suggest that GPA is associated with Band 3 in the red cell membrane as anti-GPA antibodies have been shown to decrease the rotational mobility of Band 3. A recent study [78] of the diffusion of GPA in human erythrocytes using quantum dots, however, showed that perturbation of Band 3 diffusion does not affect GPA diffusion. Our studies show that GPA can interact directly with Band 3 outside of the Ankyrin complex.

The interaction of GPA with Band 3 has two distinct physiological consequences: 1) enhanced trafficking of Band 3 from the ER to the plasma membrane, and 2) stimulation of anion transport activity [50,51]. Studies using GPA/GPB chimeras and point mutations [79] have shown that extracellular portions of GPA proximal to Arg61 that interacts with Glu658 to form the Wr antigen stimulate transport. In our simulations, we also observe interaction of residues on GPA that are proximal to Arg61 with Band 3. The extracellular interactions of GPA with Band 3 were maintained in the E658K mutant with GPA H66, H67 and F68 forming the main interactions with Band 3. The C-terminal cytoplasmic tail of GPA enhances trafficking via interaction around G701 on the cytosolic end of TM9 in Band 3. Indeed, the trafficking of the dRTA G701D mutant can be rescued by GPA [80]. In our simulations we don’t see any significant interactions of GPA with G701 but this may be due to the fact that we use a GPA with a very short cytosolic region. Furthermore, GPA mutations that interfere with dimer formation are still able to enhance Band 3 trafficking [53].

In the simulations of 64 mdAE1 dimers and 64 GPA dimers we found that GPA could bridge mdAE1 dimers forming dynamic strands of alternating GPA dimers and mdAE1 dimers. This clustering may play a key role in accumulating these two proteins at the exit sites in the ER membrane where COPII vesicles bud off to traffic newly synthesized proteins to the Golgi [81]. This may account for the stimulatory effect that GPA has on Band 3 trafficking to the cell surface. It is noteworthy that GPA can rescue the trafficking of the Southeast Asian Ovalocytosis (SAO) Band 3 deletion mutant to the cell surface [52]. This indicates that the GPA interaction with this misfolded form of Band 3 is maintained. It has been shown recently that the SAO deletion mutation changes the dynamics of the first TM helix of Band 3 in a lipid bilayer and also removes the proline induced bend [82]. In the future, we plan to use MD simulations to study the effect of the SAO deletion on Band 3 folding and its interaction with GPA.

### Limitations

It is important to consider possible limitations of the simulations used in the study. We have used the dimeric TM region of mdAE1 that was in the outward open confirmation. The cytosolic domain of mdAE1 is not present in our simulation, however, it may affect the behavior of mdAE1 e.g. it is known that the cytosolic domain interacts with the cytoskeleton. We note that whilst there are crystal structures of the separate mdAE1 and cdAE1, a complete Band 3 structure is not yet available. Therefore, there is still some uncertainty about the orientation of the cytosolic domain relative to the TM region of mdAE1. For this reason, in this study we have focused on the TM region of mdAE1 but as more structural data about the complete mdAE1 become available it would be interesting to examine how the presence of the cytosolic domain of Band 3 affects the ability of Band 3 to form tetramers and its interaction with lipids and GPA.

The use of a coarse-grained model implies some approximations on the protein and the lipids. In our CG-MD simulations we have used an elastic network model. This model restrained the protein in an outward conformation. Atomistic simulations using the last snapshot of the CG-MD systems party address this limitation, but more extended simulations or enhanced sampling techniques will be needed to address the conformational dynamics of mdAE1. Such simulation may also demonstrate how conformational changes within the protein (e.g. from outward to inward-facing state) can change its interaction with lipids. Regarding the mdAE1 interaction with GPA, we observe only the formation of the mdAE1-GPA complex, not its dissociation. This may be partly due to the timescale of the simulations. It has also been recently suggested that CG-MD simulations may exaggerate protein-protein interactions [83]. Whilst it has been suggested that the mdAE1-GPA interaction is likely to be weak, our current simulations do not allow us to give an estimate on the strength of the interactions between mdAE1 and GPA. They do provide, however, a molecular model that explains in detail the mdAE1-GPA interaction. To estimate the affinity of GPA to mdAE1 one could perform free energy calculations [84–86] although further developments of this methodology may be required to be applicable to larger protein-protein complexes such as the mdAE1/GPA complex.

## Material and methods

### Coarse-grained molecular dynamics (CG-MD) simulations

The CG-MD simulations were performed using the Martini 2.2 force field [87,88] and GROMACS [89]. For the CG-MD simulations the crystal structure of Band 3 dimer (PDB: 4YZF [11]) was converted to a coarse-grained resolution. An elastic network using a cut-off distance of 7 Å to model the protein secondary and tertiary structure was used. The elastic network restricts any major conformational change within the protein during the CG-MD simulations. We also note that prior to the conversion to the coarse-grained representation, the H_2_DIDS substrate that was included in the crystal structure was removed. Additionally, the missing unstructured regions from the crystal structure were added using Modeller [90,91].

A POPC bilayer was then self-assembled around the mdAE1 dimer (see [1]). The last snapshot from the aforementioned simulation was taken and 8 different systems were generated (see Table 1) with the protein inserted in complex asymmetric bilayers that resemble properties of the *in vivo* red blood cell plasma membrane. The exchange of lipids was done as described in [57]. All simulation systems were solvated with CG water particles and ~150 nM of NaCl was added to neutralize the systems. Prior to the production simulation all systems that did not contained SM were equilibrated for 2 ns with the protein backbone particles restrained. Systems with SM in the bilayer were equilibrated for 5 ns. Note that we have performed five self-assembly simulations and in all simulations the mdAE1 dimer interface region was occupied by lipids. In three of the simulations one lipid occupied the dimer interface during the simulations. In the other simulations two or three lipids were in the dimer interface. For that reason, for the exchange of lipids we have used one of the three systems in which a lipid was embedded in the dimer interface.

For the simulation of the mdAE1/GpA/mdAE1 complex the protein was inserted in a preformed POPC bilayer. Subsequently, two systems were generated with the protein inserted in complex asymmetric bilayers that resemble properties of the *in vivo* red blood cell plasma membrane. In one of the systems an additional restrain was added between GPA Arg61 and mdAE1 Glu658 residues. Prior to the production simulation the Band3/GpA-1 and Band3/GpA-2 systems were equilibrated for 40 ns and the Band3/GpA-3 for 90 ns with the protein backbone particles restrained (see Table 2).

To generate the large-scale systems a POPC bilayer was self-assembled around the mdAE1 dimer or the mdAE1/GPA complex. Then, the system with one protein was replicated in the xy plane using the GROMACS command genconf to create a system with 64 mdAE1 and or 64 mdAE1/GPA proteins.

The temperature for the CG-MD simulation was set to 323 K. The V-rescale thermostat [92] (coupling constant of 1.0) was used for temperature control. A Parrinello-Rahman barostat [93] (a coupling constant of 1.0 and a reference pressure of 1 bar) was used for pressure control. The integration step was 20 fs for the simulations with the Band 3 and the large-scale simulations and 10 fs for the simulations with the mdAE1/GpA/mdAE1 complex. Lennard-Jones and Coulombic interactions were shifted to zero between 9 and 12 Å, and between 0 and 12 Å, respectively.

### Atomistic molecular dynamics simulations (AT-MD)

The final snapshots of 3 repeat simulations from Band3-1 and Band3-7 simulation systems were converted to atomistic resolution as described in Stansfeld *et al*. [59]. Atomistic simulations were run using the GROMOS53a6 force field. The simulations were run for 250 ns. The V-rescale thermostat [92] and the Parrinello-Rahman barostat [93] were used for temperature and pressure control, respectively. The Particle Mesh Ewald (PME) was used to model long-range electrostatic interactions [94]. The LINCS algorithm was used to constrain bond lengths [95]. Prior to the production simulations the systems were equilibrated with the protein Cα atoms restrained for 2 ns. The simulation timestep was 2 fs and the temperature was set to 323 K. A similar approach has been used previously to study the interaction of e.g. aquaporin/PE [96], for Kir/PIP_2_ [97] and ANT/CL [98] and it yields good agreement with experimental data.

### Diffusion analysis

The diffusion analysis was performed using the open source code: https://zenodo.org/record/11827#.Whvg2racZ-U. For this analysis, the last 7 μs and 5 μs were used for the simulation with one Band 3 in the bilayer and the large-scale simulations, respectively. For all systems, the lateral diffusion was calculated. The errors for the diffusion coefficient of the systems in Table 1 is the standard deviation of the diffusion coefficient between the five replicas. The errors for the diffusion coefficient of the large-scale systems were calculated by the aforementioned code.

### Clustering analysis

Clustering analysis of the proteins was performed using the open source code: https://github.com/jhelie/cluster_prot.

### CG2AT

Simulation systems were converted to atomistic resolution as described in Stansfeld *et al*. [59].

### Analysis of the Band 3 dimer interface cavity

The calculation of the volume of the cavity in the interface of the Band 3 dimer was done using trj_cavity (https://sourceforge.net/projects/trjcavity/) [60]. Note that for this calculation we have used a ndx file that consisted the helices that form the Band 3 dimer interface. The calculation was done using either the Band 3 crystal structure or the last snapshot of the atomistic simulations. The errors for the volume of the cavity is the standard deviation of the volumes between the three replicas.

## Supporting information

S1 Fig A. Normalized contacts between mdAE1 and PIP_2_ (A), POPS (B), POPE (C), and cholesterol (D) head groups. For these histograms, the normalized contacts between the aforementioned lipids and mdAE1 from the different coarse-grained simulation systems that contained the lipids were added together. All 8 systems contained POPS and POPE lipids, 7 systems contained cholesterol and 2 systems contained PIP_2_ molecules.
(TIF)

S2 Fig A. Normalized contacts between mdAE1 and POPS and PIP_2_ molecules from the Band3-7 system. For the normalization, the number of contacts of each residue was divided by the total number of frames and the number of lipids in each simulation. This analysis demonstrates the preference of Band 3 to interact with PIP_2_ molecules. B. Convergence analysis of the interactions of mdAE1 with the lipids. The spatial distribution of PIP_2_ and cholesterol around mdAE1 in the Band3-7 system is shown for 1, 3, and 5 repeat simulations.
(TIF)

S3 Fig A. Normalized contacts between mdAE1 and PIP_2_ or cholesterol head groups (A) from the Band3_AT-1 atomistic simulations. For this analysis, the contacts from the 3 independent atomistic simulations were added together.
(TIF)

S4 Fig A, B. Contacts between the two mdAE1 monomers in our atomistic simulations (calculated for the last 30 ns of the atomistic simulations). The contacts for one of the mdAE1 proteins are shown in red and for the other proteins are shown in blue. The green vertical lines indicate the contacts found in the Band 3 crystal structure. A cut off distance of 0.4 nm was used to define a contact. The contacts are the average of 3 repeat atomistic simulations.
(TIF)

S5 Fig A, B, C, D. Normalized contacts between mdAE1 and GPA in our coarse-grained simulations with the two mdAE1 dimers and GPA. The contacts are shown for the simulation system in which we restrained the GPA Arg61/Band 3 Glu658 interaction (A, B) and for the simulation system without any restrains in the GPA Arg61/Band 3 Glu658 interaction (C, D). Because in C and D GPA interacts with only one of the monomers, the interactions from all Band 3/GPA complexes were added together.
(TIF)

S6 Fig A, B. Minimum distance between mdAE1 dimers and the transmembrane region of GPA for the simulations in which we restrained the GPA Arg61/Band 3 Glu658 interaction (A) and for the simulations without any restrains in the GPA Arg61/Band 3 Glu658 interaction (B). Note that in the simulations in which we did not include restrains in the GPA Arg61/Band 3 Glu658 interaction one of dimers diffuses away. C. Normalized contacts between mdAE1 and GPA Arg61 in the simulations without any restrains in the GPA Arg61/mdAE1 Glu658 interaction. The contacts from all systems were added together for this analysis. D. Minimum distance between mdAE1 and GPA Arg61 (black) or mdAE1 Glu658 and GPA Arg61 (red) is shown from one of the simulations.
(TIF)

S7 Fig A, B. Minimum distance between mdAE1 and GPA (A) and mdAE1 residue Lys658 and GPA residue Arg61 (B) is shown from one of the Band3/GpA-3 simulations. C. Normalized contacts between mdAE1 and GPA in our simulations with the GPA Arg61/Band 3 Glu658Lys mutation. Note that the interactions from all Band 3/GPA complexes were added together.
(TIF)

S8 Fig A. Final snapshots of the 5 repeat simulations of the Band3/GpA-1 system demonstrating the arrangement of the Band3/GPA/Band3 complex. The Band 3 monomers are shown in red and blue and the GPA in green. B. Alignment of the Band 3/GPA complexes from the Band3/GpA-1 system. The 10 different complexes are shown in different color. The four different positions of the GPA when bound on Band 3 are shown separately. C. Alignment of the Band 3/GPA complexes from the Band3/GpA-2 and Band3/GpA-3 systems. The 5 different complexes are shown in different color. Note that for clarity in B and C we show only the helical region of the GPA helix that interacts with Band 3.
(TIF)

S9 Fig A, B, C, D. Normalized contacts between mdAE1 (A, B) or GPA (C, D) and PIP_2_ or cholesterol head groups from the Band3/GpA-1 simulation. For this analysis, the contacts from the 5 independent simulations were added together. For the normalization, the number of contacts of each residue was divided by the total number of frames and the number of lipids in each simulation.
(TIF)

S10 Fig Snapshot from the start of the Band3-large (A) and Band3/GPA-large (B) simulations. The mdAE1 monomers are shown in red and blue and the GPA in green. C, D. Protein-protein interaction in A and B. The contacts are mapped onto the structure of the Band 3 dimer (for A) and of the Band 3/GPA complex (for B). Blue represents no/low number of contacts, white represents medium number of contacts and red high number of contacts. For this analysis, the contacts for all 64 individual proteins complexes in each system were added together. The contacts were calculated for the last 1 μs of the simulation to allow formation of the protein clusters. E, F. Clustering dynamics shown as the percentage of Band 3 (E) or Band 3/GPA (F) cluster size as a function of the simulation time.
(TIF)
