# Supplementary figures and images for: Interaction of the human erythrocyte Band 3 anion exchanger 1 (AE1, SLC4A1) with lipids and glycophorin A: Molecular organization of the Wright (Wr) blood group antigen
Source: PLoS Comput Biol. 2018 Jul 16;14(7):e1006284. doi: 10.1371/journal.pcbi.1006284 (PMC6080803; doi:10.1371/journal.pcbi.1006284)

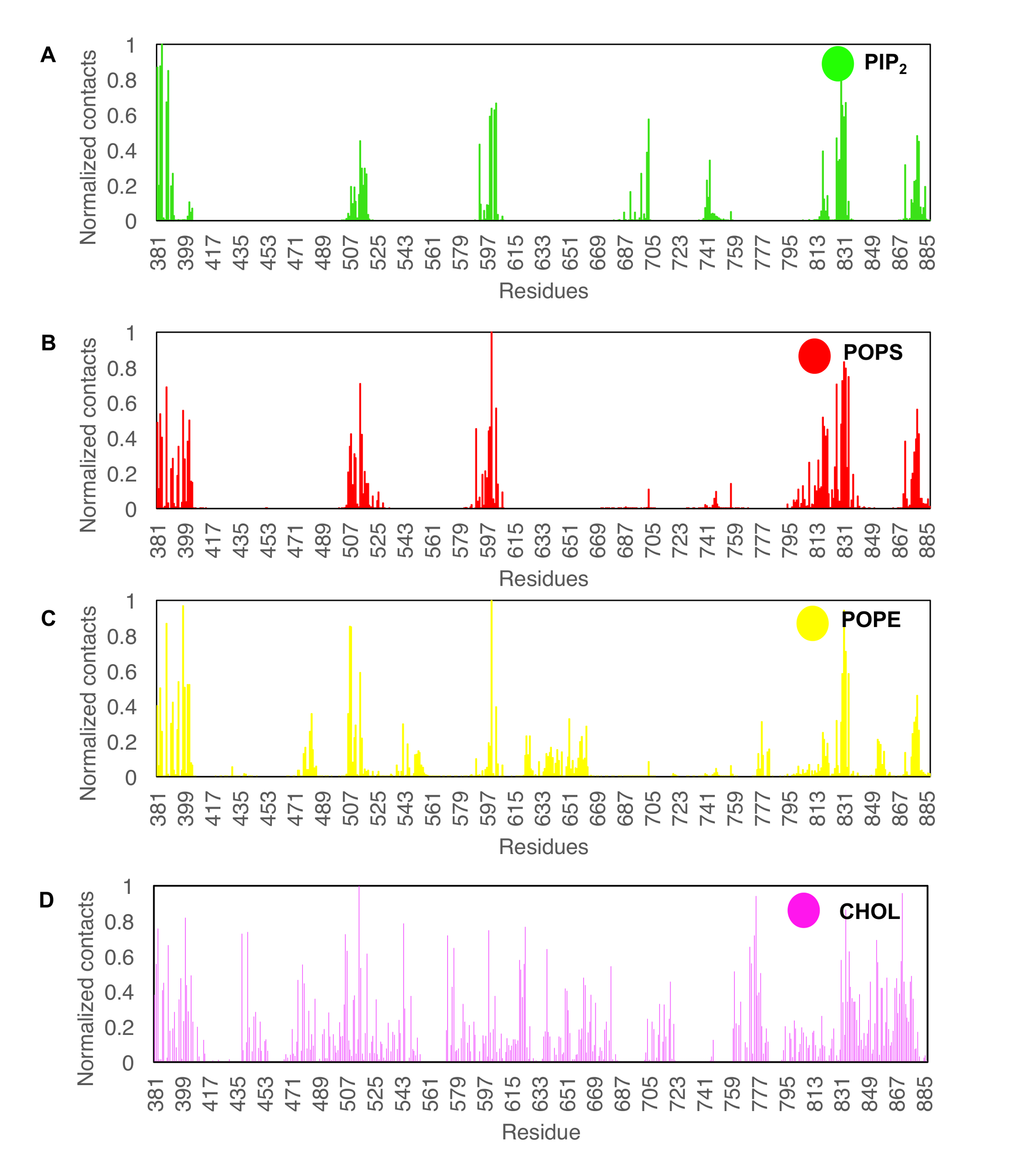

Supplement: S1 Fig — A. Normalized contacts between mdAE1 and PIP2 (A), POPS (B), POPE (C), and cholesterol (D) head groups. For these histograms, the normalized contacts between the aforementioned lipids and mdAE1 from the different coarse-grained simulation systems that contained the lipids were added together. All 8 systems contained POPS and POPE lipids, 7 systems contained cholesterol and 2 systems contained PIP2 molecules. (TIF) [file pcbi.1006284.s001.tif]

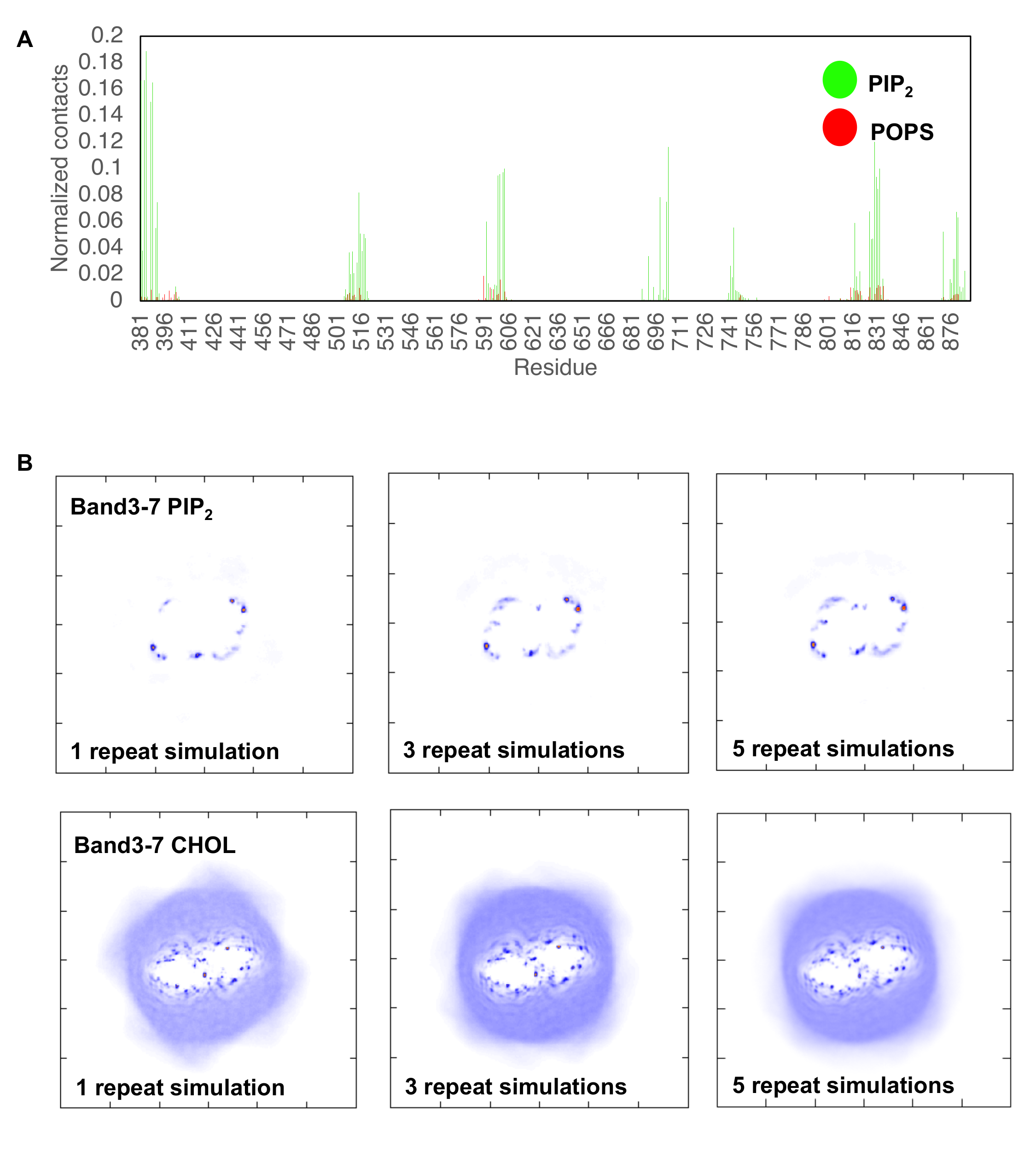

Supplement: S2 Fig — A. Normalized contacts between mdAE1 and POPS and PIP2 molecules from the Band3-7 system. For the normalization, the number of contacts of each residue was divided by the total number of frames and the number of lipids in each simulation. This analysis demonstrates the preference of Band 3 to interact with PIP2 molecules. B. Convergence analysis of the interactions of mdAE1 with the lipids. The spatial distribution of PIP2 and cholesterol around mdAE1 in the Band3-7 system is shown for 1, 3, and 5 repeat simulations. (TIF) [file pcbi.1006284.s002.tif]

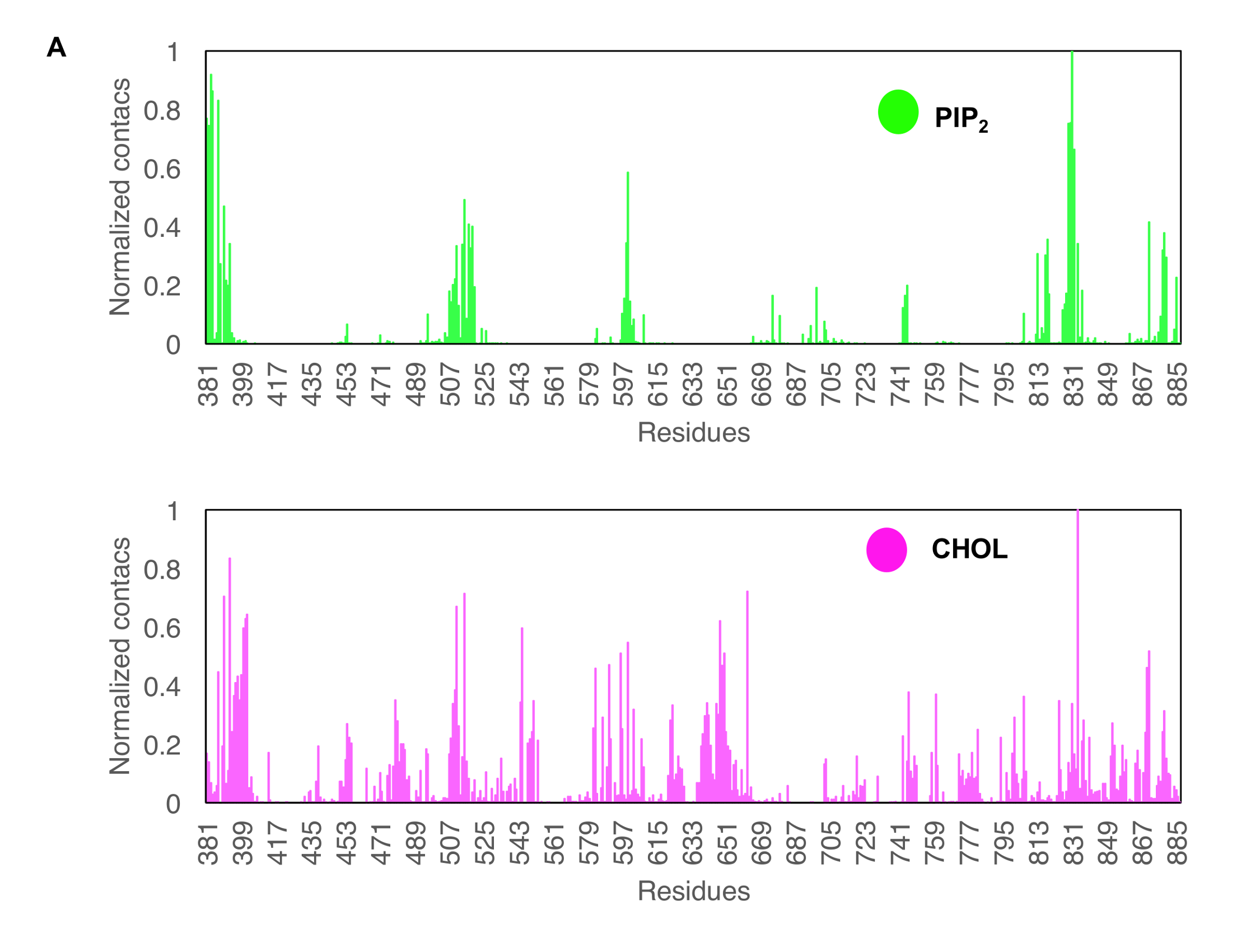

Supplement: S3 Fig — A. Normalized contacts between mdAE1 and PIP2 or cholesterol head groups (A) from the Band3_AT-1 atomistic simulations. For this analysis, the contacts from the 3 independent atomistic simulations were added together. (TIF) [file pcbi.1006284.s003.tif]

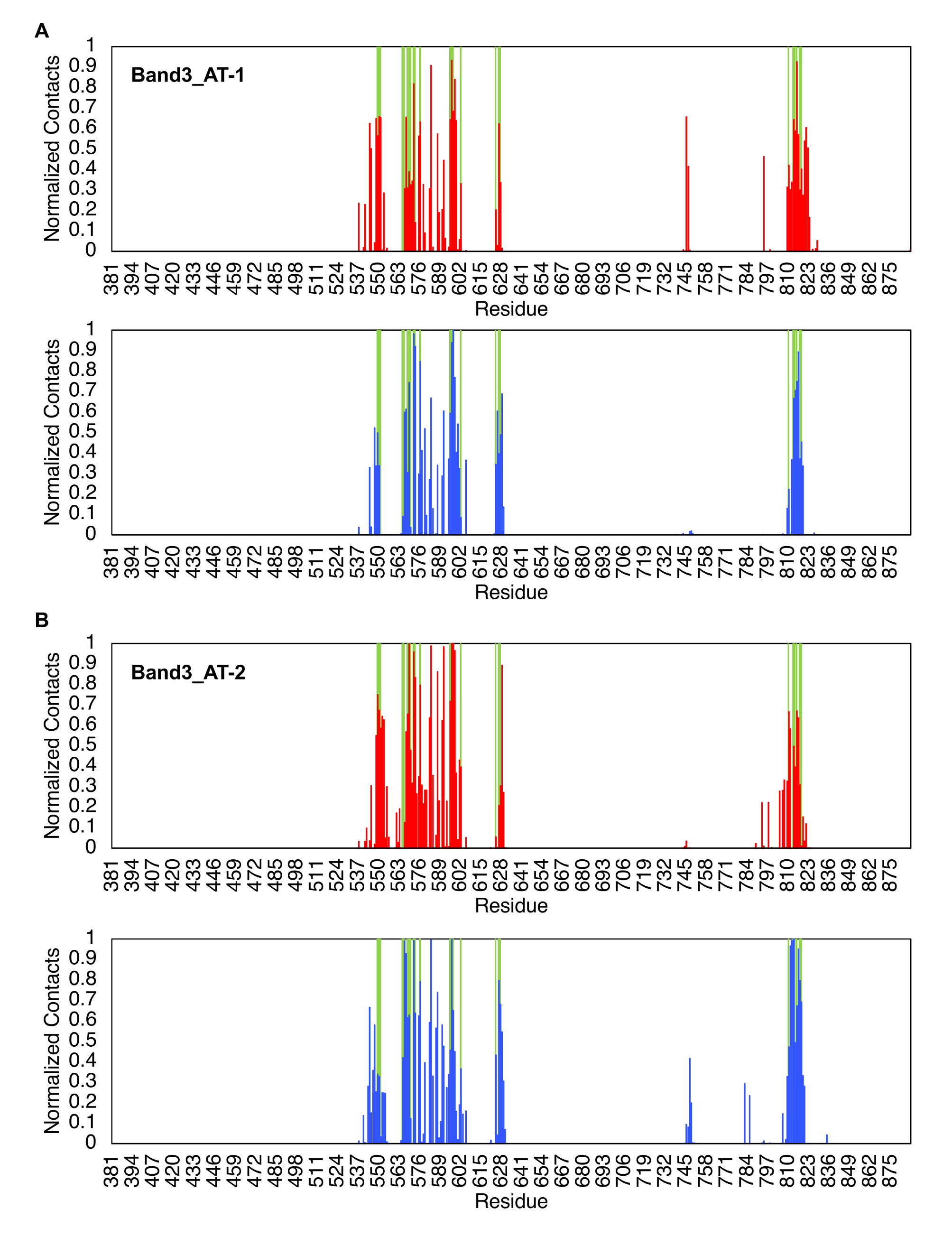

Supplement: S4 Fig — A, B. Contacts between the two mdAE1 monomers in our atomistic simulations (calculated for the last 30 ns of the atomistic simulations). The contacts for one of the mdAE1 proteins are shown in red and for the other proteins are shown in blue. The green vertical lines indicate the contacts found in the Band 3 crystal structure. A cut off distance of 0.4 nm was used to define a contact. The contacts are the average of 3 repeat atomistic simulations. (TIF) [file pcbi.1006284.s004.tif]

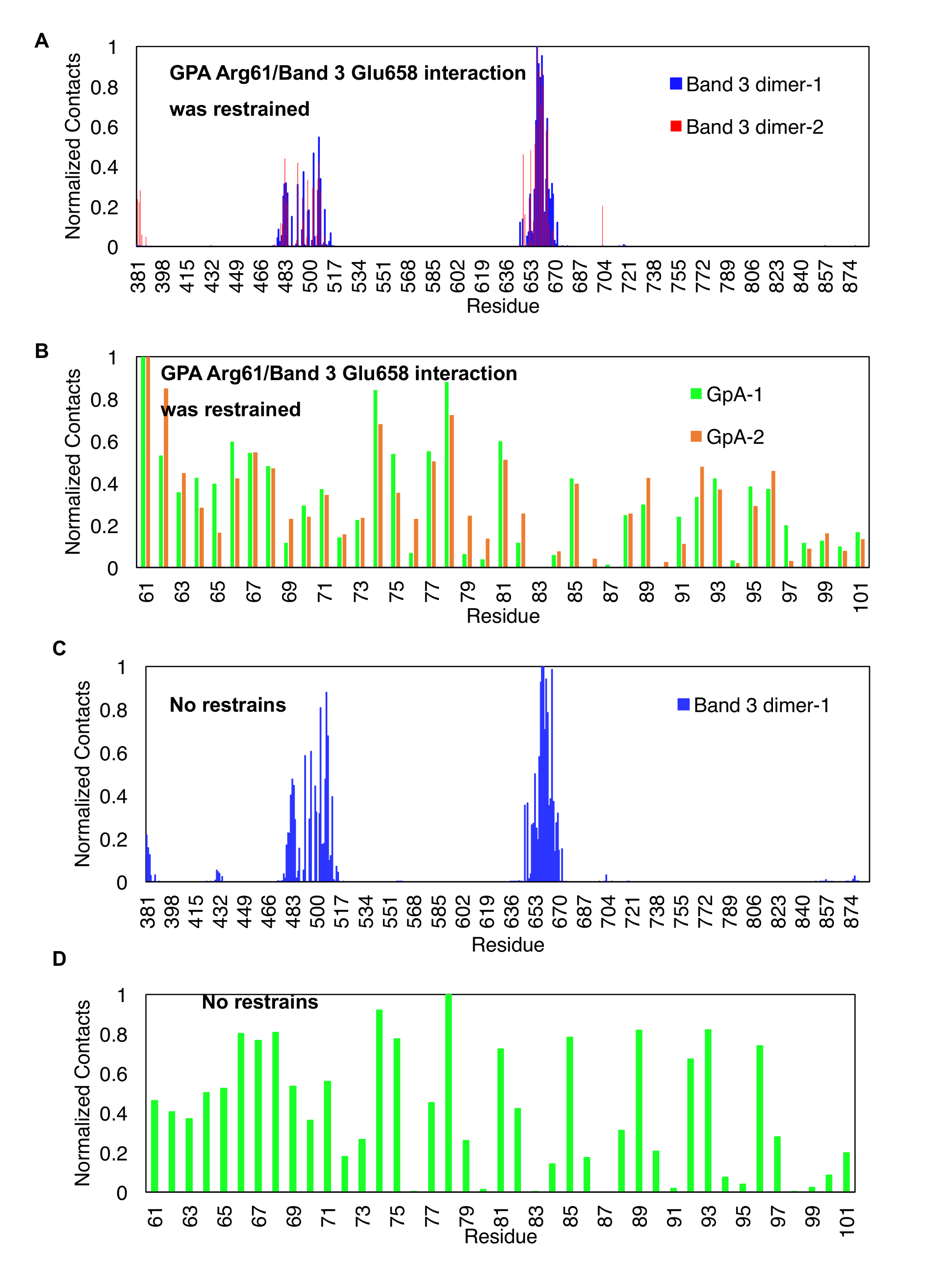

Supplement: S5 Fig — A, B, C, D. Normalized contacts between mdAE1 and GPA in our coarse-grained simulations with the two mdAE1 dimers and GPA. The contacts are shown for the simulation system in which we restrained the GPA Arg61/Band 3 Glu658 interaction (A, B) and for the simulation system without any restrains in the GPA Arg61/Band 3 Glu658 interaction (C, D). Because in C and D GPA interacts with only one of the monomers, the interactions from all Band 3/GPA complexes were added together. (TIF) [file pcbi.1006284.s005.tif]

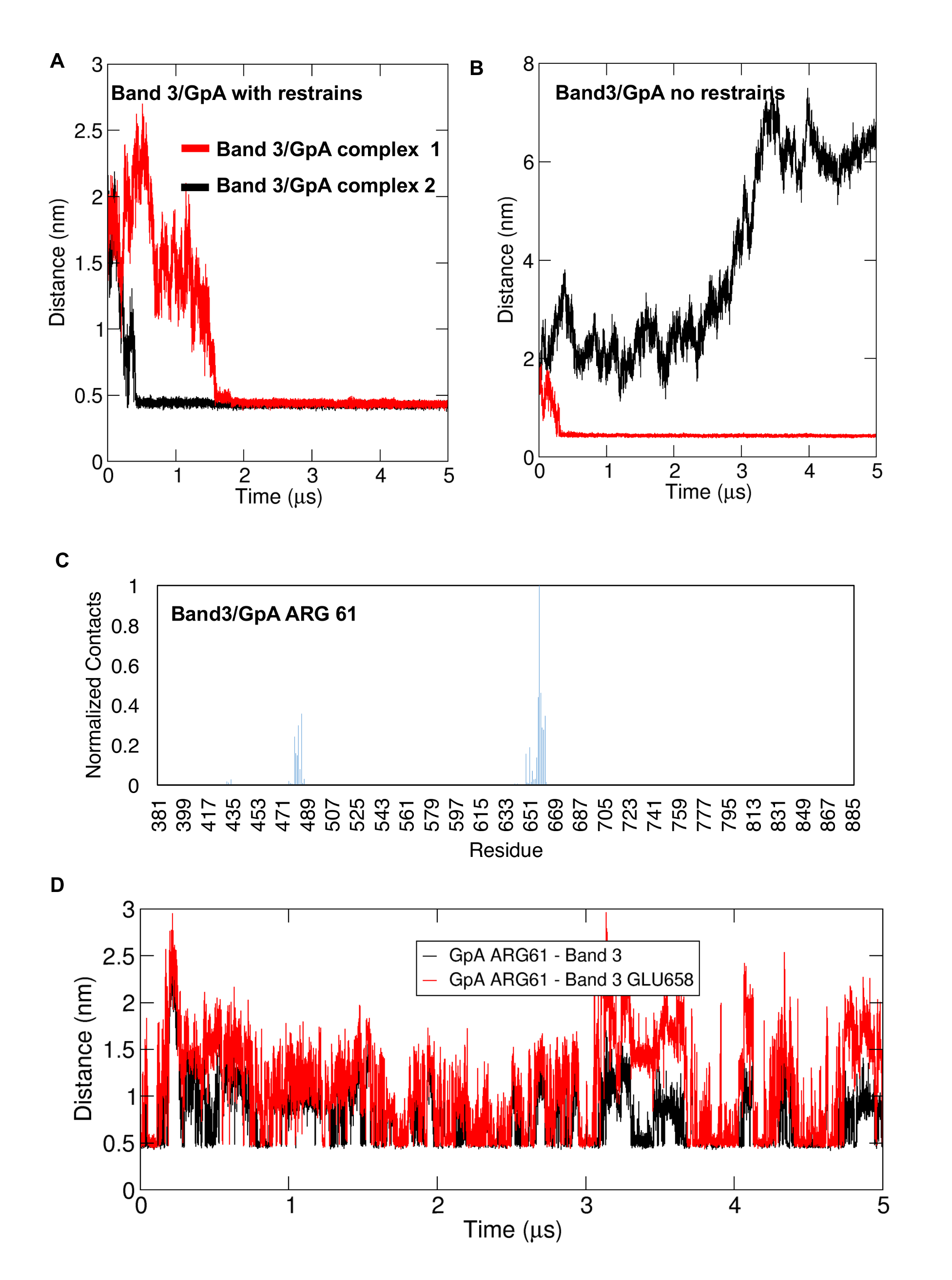

Supplement: S6 Fig — A, B. Minimum distance between mdAE1 dimers and the transmembrane region of GPA for the simulations in which we restrained the GPA Arg61/Band 3 Glu658 interaction (A) and for the simulations without any restrains in the GPA Arg61/Band 3 Glu658 interaction (B). Note that in the simulations in which we did not include restrains in the GPA Arg61/Band 3 Glu658 interaction one of dimers diffuses away. C. Normalized contacts between mdAE1 and GPA Arg61 in the simulations without any restrains in the GPA Arg61/mdAE1 Glu658 interaction. The contacts from all systems were added together for this analysis. D. Minimum distance between mdAE1 and GPA Arg61 (black) or mdAE1 Glu658 and GPA Arg61 (red) is shown from one of the simulations. (TIF) [file pcbi.1006284.s006.tif]

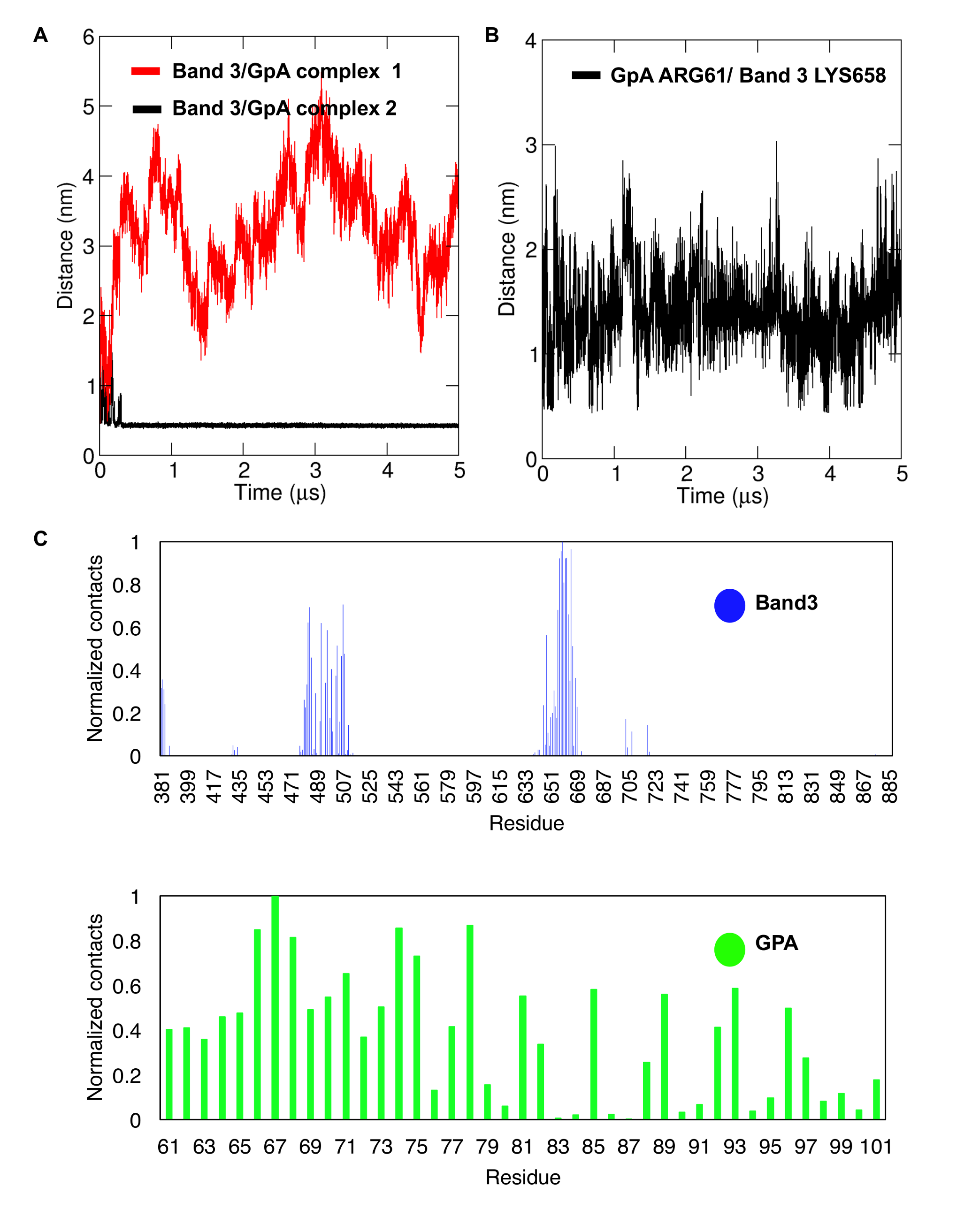

Supplement: S7 Fig — A, B. Minimum distance between mdAE1 and GPA (A) and mdAE1 residue Lys658 and GPA residue Arg61 (B) is shown from one of the Band3/GpA-3 simulations. C. Normalized contacts between mdAE1 and GPA in our simulations with the GPA Arg61/Band 3 Glu658Lys mutation. Note that the interactions from all Band 3/GPA complexes were added together. (TIF) [file pcbi.1006284.s007.tif]

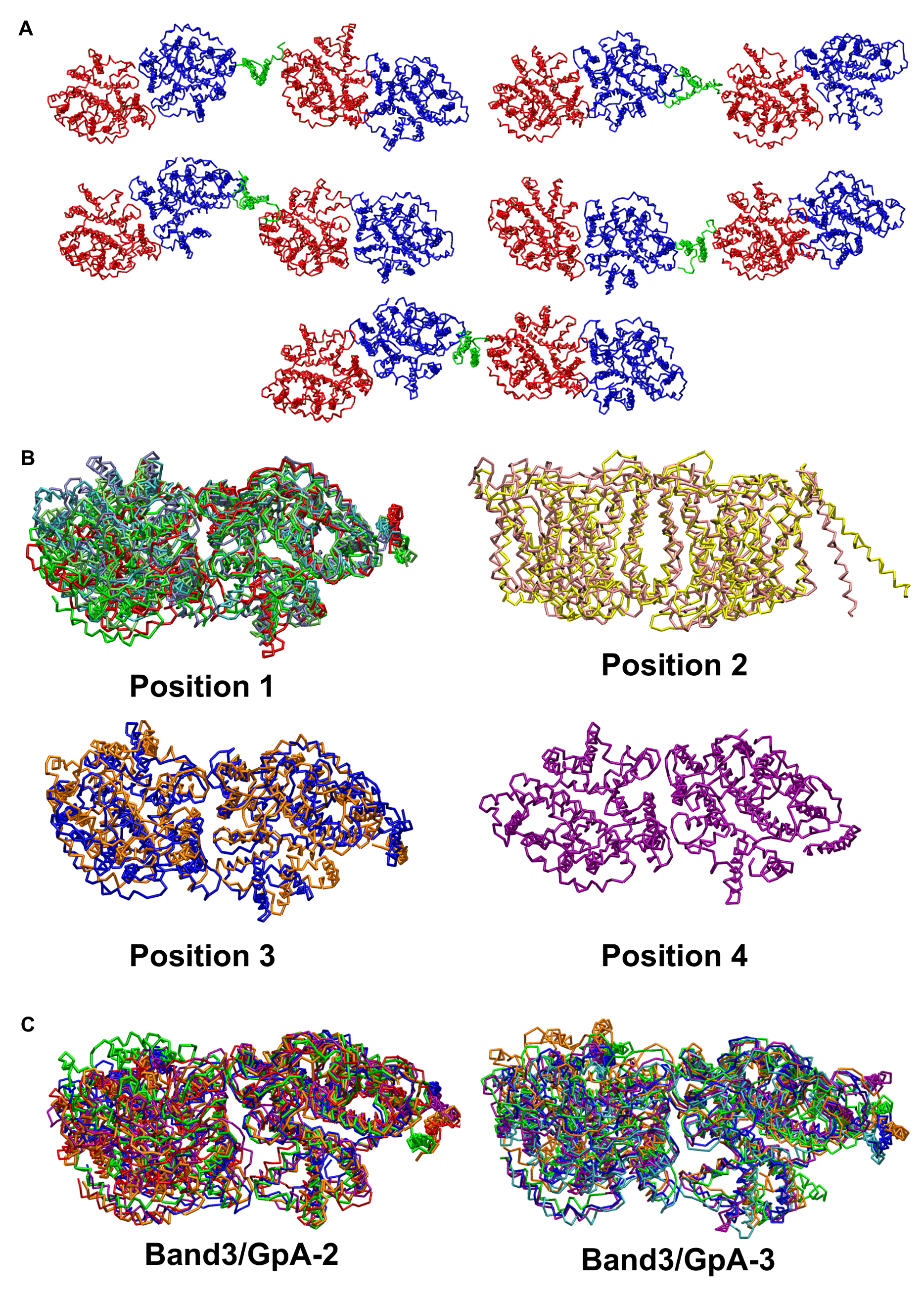

Supplement: S8 Fig — A. Final snapshots of the 5 repeat simulations of the Band3/GpA-1 system demonstrating the arrangement of the Band3/GPA/Band3 complex. The Band 3 monomers are shown in red and blue and the GPA in green. B. Alignment of the Band 3/GPA complexes from the Band3/GpA-1 system. The 10 different complexes are shown in different color. The four different positions of the GPA when bound on Band 3 are shown separately. C. Alignment of the Band 3/GPA complexes from the Band3/GpA-2 and Band3/GpA-3 systems. The 5 different complexes are shown in different color. Note that for clarity in B and C we show only the helical region of the GPA helix that interacts with Band 3. (TIF) [file pcbi.1006284.s008.tif]

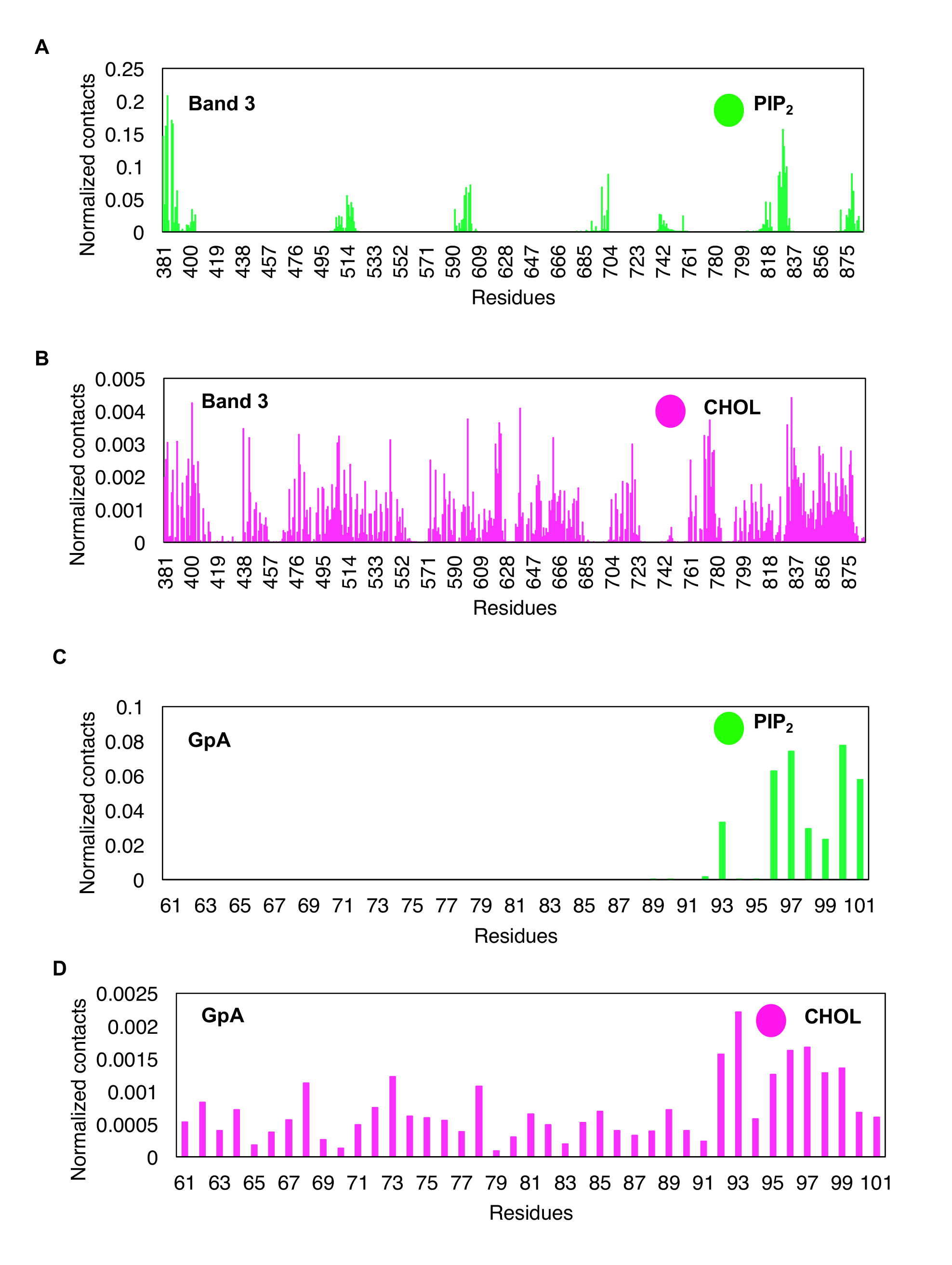

Supplement: S9 Fig — A, B, C, D. Normalized contacts between mdAE1 (A, B) or GPA (C, D) and PIP2 or cholesterol head groups from the Band3/GpA-1 simulation. For this analysis, the contacts from the 5 independent simulations were added together. For the normalization, the number of contacts of each residue was divided by the total number of frames and the number of lipids in each simulation. (TIF) [file pcbi.1006284.s009.tif]

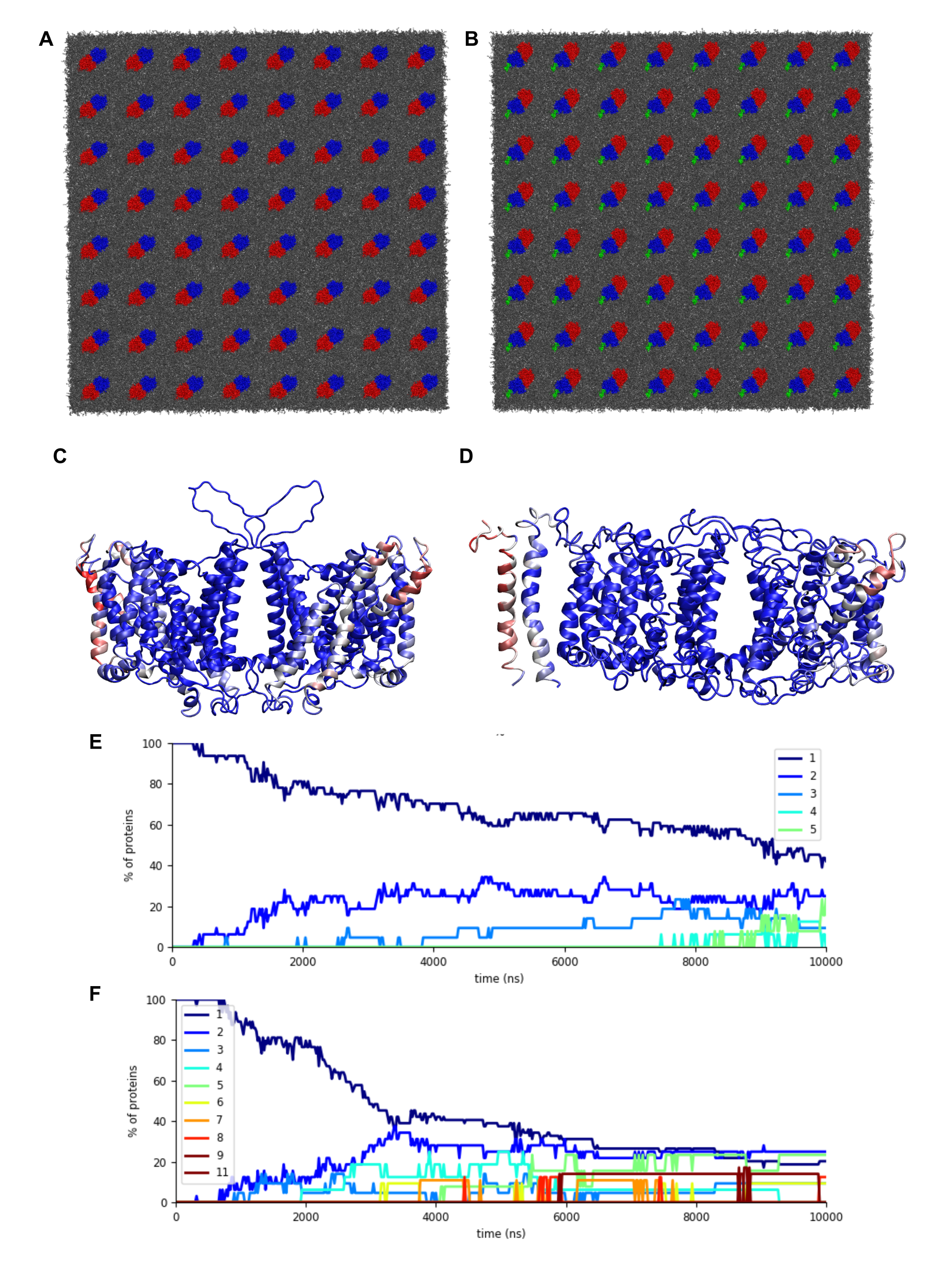

Supplement: S10 Fig — Snapshot from the start of the Band3-large (A) and Band3/GPA-large (B) simulations. The mdAE1 monomers are shown in red and blue and the GPA in green. C, D. Protein-protein interaction in A and B. The contacts are mapped onto the structure of the Band 3 dimer (for A) and of the Band 3/GPA complex (for B). Blue represents no/low number of contacts, white represents medium number of contacts and red high number of contacts. For this analysis, the contacts for all 64 individual proteins complexes in each system were added together. The contacts were calculated for the last 1 μs of the simulation to allow formation of the protein clusters. E, F. Clustering dynamics shown as the percentage of Band 3 (E) or Band 3/GPA (F) cluster size as a function of the simulation time. (TIF) [file pcbi.1006284.s010.tif]
